# Transcriptome sequencing reveals jasmonate playing a key role in ALA-induced osmotic stress tolerance in strawberry

**DOI:** 10.1186/s12870-025-06068-x

**Published:** 2025-01-11

**Authors:** Yan Zhong, Xin Wei, Jianting Zhang, Liangju Wang

**Affiliations:** 1 College of Horticulture, Nanjing Agricultural University, Nanjing, 211800 China; 2 Modern Agricultural Analysis and Testing Center, Nanjing Agricultural University, Nanjing, 211800 China

**Keywords:** 5-Aminolevulinic acid (ALA), Jasmonates signals, Osmotic stress, RNA-sequencing, Strawberry

## Abstract

**Background:**

Strawberry (*Fragaria* × *annanasa* Duch.) is an important economic fruit worldwide, whose growth and development are often hindered by water deficiency. 5-Aminolevulinic acid (ALA), a natural plant growth regulator, has been suggested to mitigate the osmotic damages by promoting root water absorption, osmotic adjustment, photosynthetic capacity, and antioxidant improvement. However, the regulatory mechanism remains unclear.

**Results:**

In the current study, the underlying mechanism by determination of various physiological indices, as well as transcriptome sequencing and the weighted gene correlation network analysis (WGCNA) of 10 mg L^− 1^ ALA treated strawberry leaves and roots stressed by 20% polyethylene glycol 6000 (PEG) treatment. The findings indicated that ALA enhanced osmotic stress tolerance reflected by enhancing relative water content (RWC), root development, gas exchange parameters and antioxidant enzyme activities, and decreasing the leaf H_2_O_2_ and malondialdehyde (MDA) content. Transcriptome analysis showed that the differentially expressed genes (DEGs) stimulated by exogenous ALA were mostly associated with the secondary biosynthesis and hormones signaling pathways, especially jasmonates (JAs). The JA derivative (+)-7-iso-jasmonoyl-L-isoleucine (JA-Ile) was found to be elevated in the strawberry leaves and roots treated with ALA under PEG stress. Additionally, exogenous methyl jasmonate (MeJA) alleviated osmotic stress damages similarly to ALA, while its synthesis inhibitor diethyldithiocarbamate (DIECA) led to adverse effects on strawberries, which can be relieved by further additional application of ALA.

**Conclusions:**

Theses findings suggest that JAs can act as the necessary signaling molecules involved in ALA-improved osmotic stress tolerance networks. This provides a new insight for further study on how ALA can help plants cope with water stress.

**Supplementary Information:**

The online version contains supplementary material available at 10.1186/s12870-025-06068-x.

## Background

Strawberry (*Fragaria* × *ananassa* Duch.) is one of the well-received fruit crops worldwide because of its typical flavor, attractive color, and abundant nutritional value, such as sugars, minerals, phenolic compounds, and vitamin C [1]. China is the largest producer and exporter of strawberry worldwide [2]. However, the environmental conditions lead to a lot of challenges in strawberry production due to the shallow root systems and intensive leaf transpiration. Therefore, water deficiency is one of the adverse factors influencing the growth and development of plants as well as the yield and quality of fruits [2, 3]. Drought conditions exert adverse impacts on physiological and biochemical responses of strawberries, such as chlorophyll degradation, stomatal closure directly leading to of photosynthesis suppression, reactive oxygen species (ROS) imbalance, which impose constraints on their growth, yield and quality [3–5]. Thereby, drought tolerance improvement and underlying mechanisms are crucial to meet the needs for stable-yield and high-quality strawberry production.

5-Aminolevulinic acid (ALA), a non-protein amino acid, is a new environmentally-friendly plant growth regulator, that exists as the key biosynthetic precursor of all tetrapyrroles in plants and animals, such as chlorophyll, heme, and cytochrome [6]. ALA is of great benefit to plant growth and development, fruit quality, and even the improvement of stress-tolerance against various harmful ecological factors. ALA has been widely proven to contribute to drought tolerance by means of alleviating stress injuries, including biomass, photosynthetic capability and nitrogen assimilation enhancement, oxidative damage reduction, osmotic substance and essential mineral accumulation [7, 8]. For example, ALA restrains the drought injuries on photosynthesis through improving the chlorophyll content, net photosynthetic rate and the tissue water content in strawberry [9]. Under water deficit condition, ALA also enhances the antioxidant enzyme activity such as superoxide dismutase (SOD), peroxidase (POD), catalase (CAT) and ascorbate peroxidase (APX) to eliminate the ROS [9–12], as well as the osmolytes for osmotic adjustment [13, 14] in many commercial crops, such as the strawberry. ALA was found to upregulate expression of *psb* genes of photosystem II (PSII) reaction center in drought-stressed strawberry [9], and *P5CS* and *P5CR* genes for proline biosynthesis in salt-stressed tomato [15]. Notably, the mechanism by which exogenous ALA maintains strawberry water balance is revealed through its acceleration on leaf stomatal conductivity and transpiration rate, in combination with the enhancement on the aquaporin gene expressions [9]. It is conducive to water absorption and transport in drought-stressed strawberries [9]. However, few studies focusing on molecular mechanisms of ALA regulating the alleviation between plant growth and survival under osmotic stress have been systematically reported. It is known that ALA can induce the biosynthesis and accumulation of jasmonic acid (JA), which is associated with ALA-mediated cold tolerance of tomato [16] and salt resistance of *Populus wutunensis* [17]. JA and its derivatives, methyl jasmonate (MeJA) and (+)-7-iso-jasmonoyl-L- isoleucine (JA-Ile), have an intense cross-talk with other plant hormones and secondary metabolites [18–20]. Therefore, JAs play pivotal roles not only in plant growth and development, but also in the response to biotic and abiotic stresses [21]. In JAs signaling and transduction, JA-Ile promotes the combination of JA ZIM-domain (JAZ) repressor protein with JA receptor Skp/Cullin/F-box CORONATINE INSENSITIVE1 (SCF^COI1^) protein, and subsequently, the JAZ degradation leads to the release of transcription factors (TFs) for the JA cascade of plants [22–25].

Up to now, a few studies have been conducted to reveal the mechanisms of exogenous ALA on anthocyanin biosynthesis in apple [26], tetrapyrrole metabolism in rice [27] and salt tolerance improvement in *Populus wutunensis* [17] at the transcriptomic level. However, few report has discovered the ALA-stimulating molecular mechanisms in response to osmotic stress through transcriptome sequencing and systematically analysis. Therefore, this study was performed to identify the key genes and their networks involved in ALA-improved osmotic stress tolerance of strawberries. Here, the samples were collected from strawberries treated with exogenous ALA under 20% polyethylene glycol 6000 (PEG) stress for the determination of relative water content (RWC), gas exchange parameters, H_2_O_2_ content, antioxidant enzyme activity and other indicators, especially for RNA-sequencing (RNA-seq) and differentially expressed genes (DEGs) filtering and analyzing. Besides, exogenous MeJA and its biosynthesis inhibitor diethyldithiocarbamate (DIECA) were further applied to identify the JAs role in the process of ALA-induced osmotic stress tolerance of strawberries. Our findings demonstrated that ALA-JAs participated in osmotic stress tolerance enhancement of strawberries, providing a theoretical basis for ALA application of in strawberry production.

## Methods

### Plant materials and treatments

The octoploid cultivated strawberries (*F.* × *annanasa* Duch cv. ‘Benihoppe’) cultivated by the Jiangsu Academy of Agricultural Sciences, were grown in a plant growth chamber with photosynthetic photon flux densities of 300 µmol m^− 2^ s^− 1^, the light/dark photoperiods of 16 h/8 h, and a temperature of 25 ± 1℃ and a humidity of 65%. The five-leaf-old strawberries were planted in plastic cups filled with peat soil, vermiculite, and perlite (volume ratio = 4: 2: 1), were applied with 200 mL of ^1^/_2_ Hoagland nutrient solution [28] every three days. Following a nine-day reestablishment, the strawberries underwent three types of treatments: well watered with ^1^/_2_ Hoagland nutrient solution (control), osmotic stress (a solution containing 20% PEG in ^1^/_2_ Hoagland nutrient solution), and osmotic stress plus ALA treatment (^1^/_2_ Hoagland nutrient solution containing 20% PEG and 10 mg L^− 1^ ALA). The plants of three treatments were irrigated with 200 ml of their corresponding solutions every three days, respectively. In our preliminary experiment, the PEG concentrations ≤ 15% was not enough high for osmotic stress simulation on strawberries, and 20% was selected as an appropriate concentration. The ALA concentration was referred to our previous study [9]. Their leaves and roots were collected at the relative early stage (2, 4, and 6 days) after each treatment and frozen in liquid nitrogen. They were all biologically repeated three times for the following physiological biochemical and RNA sequencing analysis to discover the relatively earlier and more sensitive responses.

Additionally, another group experiment was implemented for six days: ^1^/_2_ Hoagland nutrient solution (control), ^1^/_2_ Hoagland nutrient solution containing 20% PEG, ^1^/_2_ Hoagland nutrient solution containing 20% PEG + 10 mg L^− 1^ MeJA, ^1^/_2_ Hoagland nutrient solution containing 20% PEG + 20 mg L^− 1^ DIECA, and ^1^/_2_ Hoagland nutrient solution containing 20% PEG + 20 mg L^− 1^ DIECA + 10 mg L^− 1^ ALA. The concentrations of MeJA and DIECA used here were within the ranges applied on plants in response to various stresses [29–31]. The leaves and roots were sampled at the sixth day after treatments for triple repeated physiological measurements.

### Determination of root traits and relative water content (RWC)

The lengths, surface areas, volumes, and diameters of the strawberry roots were measured using a Hewlett Packard scanner controlled by Win-RHIZO based on the study of Sial et al. [32]. The root vitality was determined by 2,3,5-triphenyltetrazolium chloride method [33]. The clean sample weights were determined as fresh weights (FWs). For measurement of the saturated weights (SWs), the samples were immersed in sterile water for 5 h, then the plant tissues were wiped of surface moisture and the culture matrix was filtrated out excess moisture. These wet samples were dried at 70 °C for 48 h for dry weights (DWs) determination. The FWs, DWs and SWs of treated strawberries or culture matrix were measured to calculated the RWC = (FW − DW)/(SW − DW) × 100%, according to the method provided by Meher et al. [34]. All the examinations were repeated for three times.

### Measurement of gas exchange parameters

The gas exchange parameters of well-developed strawberry leaves were measured simultaneously using a Li-6800 photosynthesis system (LI-COR, Lincoln, NE, USA) at 9:00–11:00 a.m [35]. The conditions included an atmospheric CO_2_ concentration of 400 µmol mol^− 1^, a leaf chamber temperature of 22℃, a light intensity of 1800 µmol m^− 2^ s^− 1^ and 50% humidity. Fifteen leaves of each treatment were measured for accuracy.

### Measurement of chlorophyll content

The content of total chlorophyll, chlorophyll a, and chlorophyll b was measured according to the method reported by Meher et al. [34], by using the Multimode Microplate Reader at wavelengths of 663 nm and 645 nm. All determinations of chlorophyll content were carried out for three repetitions.

### Measurement of physiological and biochemical indices

The nitrogen blue tetrazole, guaiacol and permanganimetric methods were used to measure the antioxidant enzyme activity of superoxide dismutase (SOD), peroxidase (POD) and catalase (CAT), respectively [36]. Determination of the H_2_O_2_ content was following the method of Ferguson et al. [37]. The malondialdehyde (MDA) content was examined by using the thiobarbituric acid (TBA) method [37]. The free proline content was estimated by acidic-ninhydrin method as previously described [38]. All the measurements were biologically repeated three times.

### Determination of endogenous JAs content

The determination of endogenous jasmonic acid like hormones was performed according the method proposed by Pan et al. [39]. Each sample of 500 mg fresh strawberry leaves and roots was respectively weighed and frozen in liquid nitrogen, which was immediately ground in the grinding machine, transferred to a 10 mL centrifuge tube, and 2 mL of pre-cooled extraction solution was added (isopropanol: ultrapure water: concentrated HCl = 2: 1: 0.002, v/v/v, each sample containing 10 ng dihydrojasmonic acid as the internal standard). The centrifuge tube was rotated in a 4℃ shaker at 150 rpm in dark for one hour. Each tube was then filled with 4 mL of pre-cooled chromatographically pure dichloromethane, continuing to shake in dark to mix for an hour. Finally, they were centrifuged at 4℃ 6000 × g for 10 min. Four milliliters of the lower layer solution were pipetted into a new centrifuge tube, concentrated with a nitrogen blower. Then, 400 µL pre-cooled spectrum pure methanol was used to dissolve the sample, centrifuge with 6 000 × g at 4℃ for 10 min. All the supernatant was transferred to a new 1.5 mL centrifuge tube, and concentrated with a nitrogen blower until completely dry. Subsequently, the sample was redissolved in 50 µL pre-cooled pure spectrum methanol, and centrifuged with 12,000 × g at 4 ℃ for 15 min, and the supernatant was measured by high pressure liquid chromatography-mass spectrometry (HPLC-MS). Each measurement was performed three biological replicates.

### RNA extraction and transcriptome sequencing

The leaves and roots underwent control, PEG, and PEG + ALA treatments for 2, 4, and 6 days were sampled for RNA extraction (Trizol reagent kit, Invitrogen, Carlsbad, CA, USA), respectively. The total RNA was tested by an Agilent 2100 Bioanalyzer (Agilent Technologies, Palo Alto, CA, USA) and electrophoresis, in which the high-quality mRNA was enriched by Oligo (dT) beads, and reverse transcribed into cDNA. Following ligation using Illumina sequencing adapters and PCR amplification, the products were sequenced using Illumina Novaseq6000 by Gene Denovo Biotechnology Co. (Guangzhou, China). Each sample was biologically replicated three times.

### Data analysis

The raw data were filtered to remove adapters, low quality reads with more than 50% of low quality bases (Q-value ≤ 20) and reads with more than 10% unknown nucleotides (N) by using fastp v0.18.0 with the parameters of ‘-q 20 -u 50 -N 15 –I 50’ [40], and then mapped to ribosome RNA (rRNA) database by Bowtie2 v2.2.8 with the parameter of ‘--local’ [41]. After eliminating the rRNA mapped reads, the obtained clean reads were mapped to *F. x ananassa* ‘Camarosa’ Genome v1.0.a1 (GDR, https://www.rosaceae.org/) using HISAT2.2.4 with the default parameters [42]. A reference-based method was performed to assemble the mapped reads by StringTie v1.3.1 with the parameter of ‘-f 0.3’ [43, 44], and the fragment per kilobase of transcript per million reads (FPKM) values were computed by using RSEM software with the default parameters [45].

The differentially expressed genes (DEGs) were filtered by using DESeq2 package [46] under the parameters of absolute fold change (|log_2_FC|) ≥ 1 and false discovery rate adjusted *P* value < 0.05. The expression heatmap of DEGs were visualized by the pheatmap (https://cran.r-project.org/web/packages/pheatmap) and ggplot2 (https://cran.r-project.org/web/packages/ggplot2/) packages of R procedure based on their standardized FPKM values. The DEGs among different treatments were also detected for the gene ontology (GO, http://geneontology.org) enrichment [47] and Kyoto encyclopedia of genes and genomes (KEGG) enrichment [48]. The expression patterns clustering of DEGs under different treatments were conducted by using Mfuzz package [49], in which genes exhibiting the similar expression pattern were classified into the same cluster based on the membership values.

In addition, the weighted gene correlation network analysis (WGCNA) [50] was performed to divide the DEGs into different modules by R scripts. The analysis and visualization of GO, KEGG, and co-expression network were also depending on R scripts. Therefore, the hub genes of the modules were identified according to the connectivity and weight values.

### Analysis of gene expression by real-time quantitative PCR (RT-qPCR)

Total RNA of strawberry tissues was extracted using the RNA extraction kit (Biofit Biotech, Hangzhou, China), followed by removing DNA and reverse transcription using TranScript One-Step gDNA Removal and cDNA Synthesis Supermix (Transgen Biotech, Beijing, China). Subsequently, RT-qPCR assays were carried out by using the SYBR^®^ Premix Ex Taq TM kit (Tli RNaseH Plus, TaKaRa, RR420A), with the cDNA as the template and *FaActin* as the reference gene. The relative expression of genes was calculated based on the comparative CT (2^−ΔΔCT^) method [51]. The specific primers (Table S1) of the genes used here were designed by Primer Premier 5.0 software. Three repetitions were created in the RT-qPCR tests.

### Statistics analysis

All measurements were biological repeated for at least three times. Statistical analysis was carried out using SPSS 20.0 statistical program. Significant differences (*P* < 0.05) of data were compared among treatments by analysis of variance (ANOVA) and Duncan’s multiple range test. Graphs were generated in Excel 2016 and Origin Pro 7.5.

## Results

### ALA enhances the osmotic stress tolerance of strawberries

**Fig. 1:**
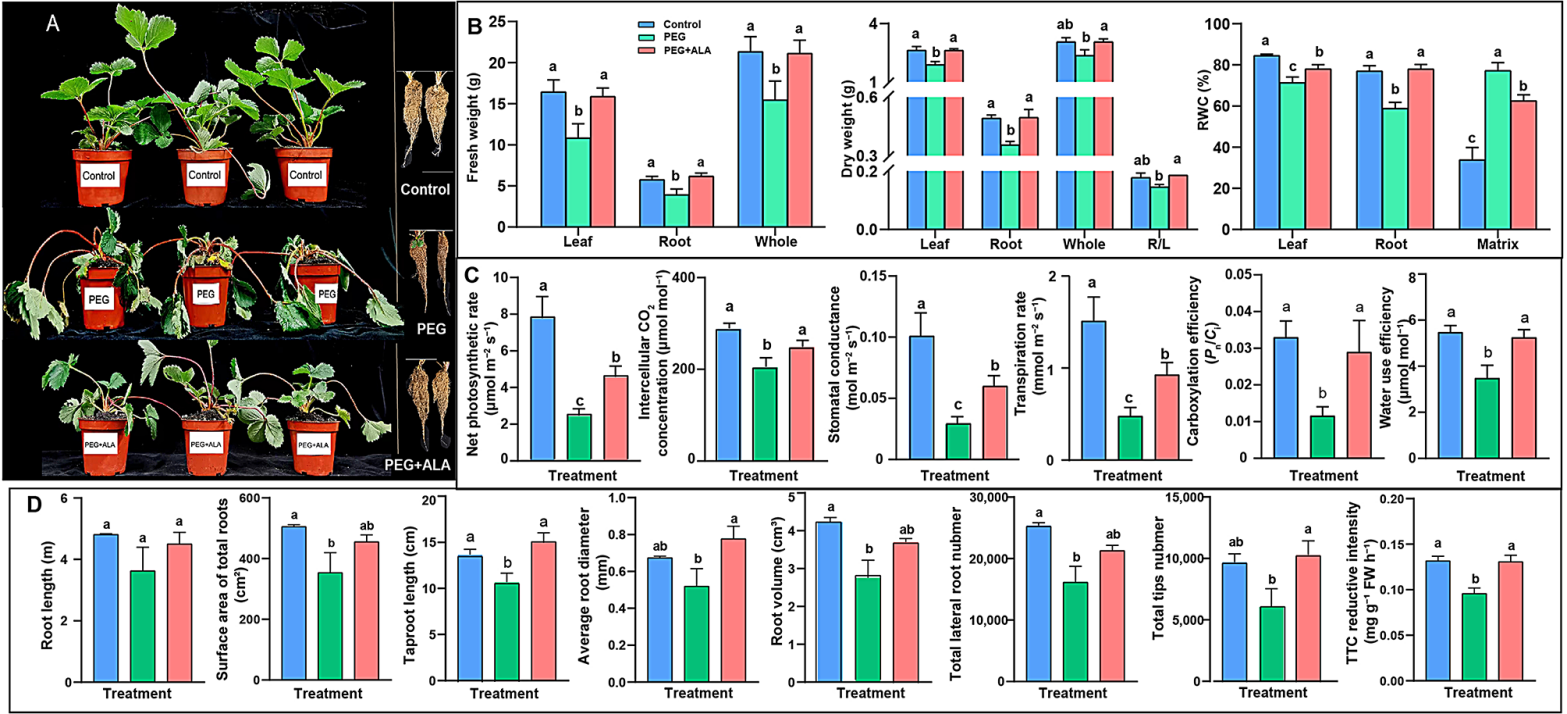
Effects of ALA on osmotic stress tolerance of strawberries after PEG treatment for six days. **A**: The phenotypes of strawberries under different treatments. **B**: The fresh weights, dry weights and RWC of strawberries and culture matrix/substrate. **C**: The gas exchange parameters of strawberry leaves. **D**: The indices related to root traits under treatments. The same lowercases in each index or tissue represent no significant difference at *P* = 0.05 level

The PEG-induced osmotic stress caused lots of injuries on strawberries compared with the control after treatment for six days, such as the dehydrated plant, wilted leaves, weakening and browning root systems. However, PEG + ALA led to mitigation of these damages, including smooth leaves, forceful plants, and more biomass of strawberries than that of the PEG only (Fig. 1A). The remission effect of ALA on drought-injured strawberries was also reflected by the RWC, gas exchange parameters and various root trait indices. The fresh weights, dry weights and RWC of different strawberry tissues exposed to osmotic stress were significantly lower than those under control condition, which were significantly improved by PEG + ALA (Fig. 1B, *P* < 0.05). Conversely, the RWC of culture matrix was significantly increased by PEG stress compared with that of the control, but decreased by the addition of ALA (*P* < 0.05). These indicate that ALA is beneficial for plant to absorb water from the high osmotic matrix to maintain water homeostasis. Furthermore, PEG stress severely affected the photosynthetic gas exchange parameters (Fig. 1C), causing the net photosynthetic rate (*P*_n_), intercellular CO_2_ concentration (*C*_i_), stomatal conductance (*G*_s_), transpiration rate (*T*_r_), instantaneous carboxylation efficiency (*P*_n_/*C*_i_) and water use efficiency decreased by 68%, 9%, 71%, 68%, 65% and 36% compared with those of the control, respectively. However, application of ALA significantly enhanced these parameters by 86%, 12%, 105%, 94%, 150% and 51% in comparison with those of PEG stress alone, respectively (*P* < 0.05). For the root systems, different treatments did not significantly affect root length, however, ALA significantly depressed the osmotic stress impairment on taproot lengths, average root diameters, total tips numbers, and root activity (TTC reductive intensity) of strawberries (Fig. 1D, *P* < 0.05). Additionally, the promotive effects of ALA on surface area of total roots, root volume, and the numbers of total lateral roots can be observed, yet, they were not statistically significant at *P* = 0.05. Based on these, ALA application is a great help to alleviate the harmful effects caused by PEG-induced osmotic stress on strawberry growth and development, as well as leaf photosynthetic capacity.

### Effects of ALA on the antioxidant capability in strawberries under PEG stress

**Fig. 2:**
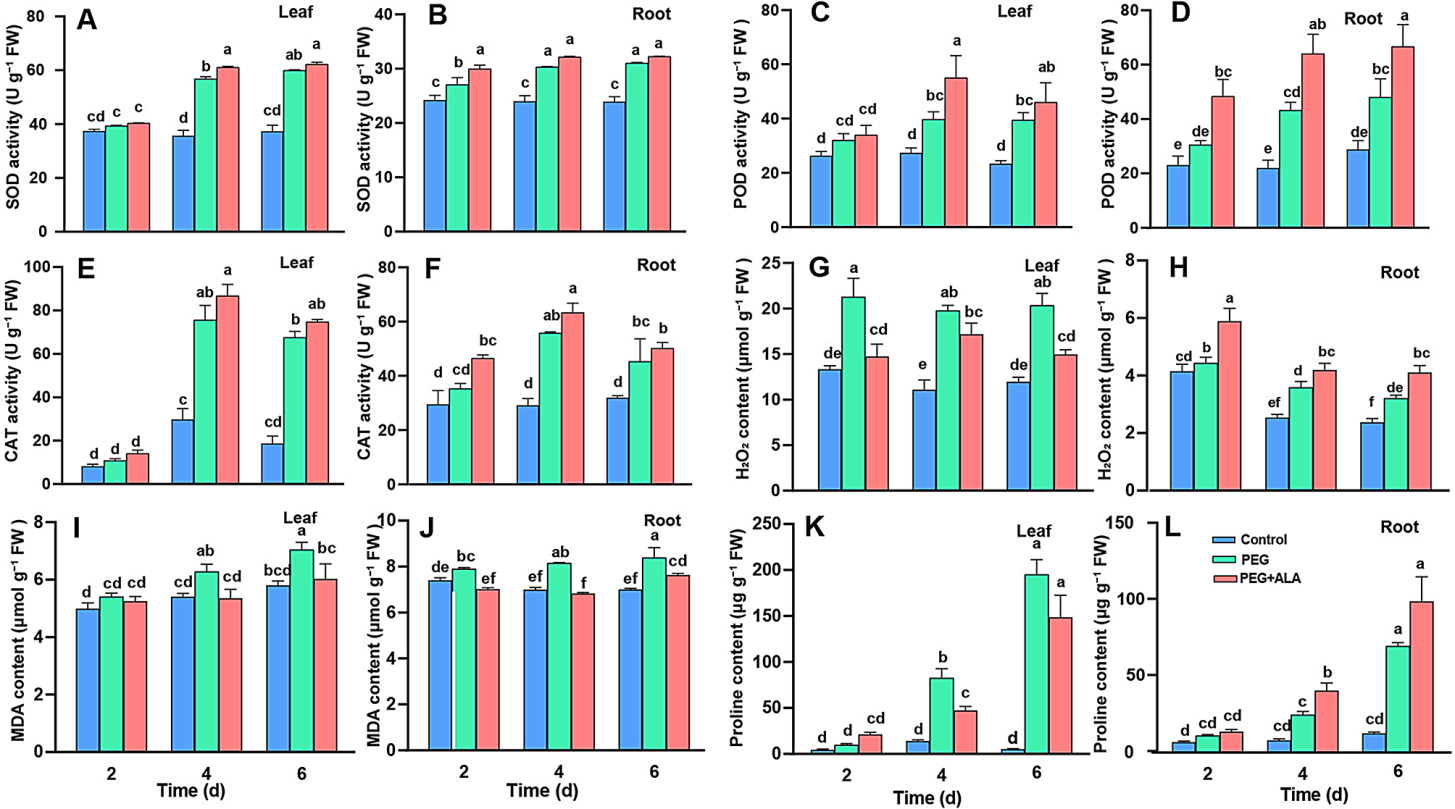
The activities of SOD (**A**, **B**), POD (**C**, **D**) and CAT (**E**, **F**), and the content of H_2_O_2_ (**G**, **H**), MDA (**I**, **J**) and proline (**K**, **L**) in strawberry leaves and roots, respectively, under different treatments. The same lowercases in each panel represent no significant difference at *P* = 0.05 level

The activities of antioxidant enzymes, including SOD, POD and CAT in the leaves and roots of strawberries were gradually increased after PEG treatment (*P* < 0.05), which were further promoted by exogenous ALA (Fig. 2A-F). Relatively, the SOD and CAT activities were more responsive to PEG than to ALA treatment, while the POD activities were more responsive to ALA treatment than those of SOD and CAT. On the other hand, the enzyme activities in the roots were also responsive to PEG or/and ALA treatment, with a similar pattern of leaves. It seems that the SOD and POD activities in roots were more quickly stimulated by exogenous ALA than those in leaves, which suggests that ALA provided faster assistance to antioxidant activity in roots than in leaves when exposed to PEG. Furthermore, ALA + PEG treatment preferred stimulating SOD and POD activities to those of CAT for sensitive antioxidant response in strawberries against osmotic stress.

H_2_O_2_ is a main reactive oxygen species, whose content was increased significantly in leaves and roots after PEG stress, but diversely influenced by ALA in different tissues (Fig. 2G-H). In the leaves, ALA inhibited the H_2_O_2_ increases, while consistently promoted the levels in the roots when plants were stressed by PEG (*P* < 0.05). The tissue-specific responses imply that ALA may regulate the H_2_O_2_ levels to play different roles in different tissues of strawberry plants confronted with osmotic stress.

MDA, the lipid peroxidation product, was increased by PEG, which continued to accumulate in leaves and roots along with the stress process (Fig. 2I-J). When ALA treatment was performed, the significant inhibition of MDA accumulation was distinctly exhibited in leaves with both 15% decreases at four and six days, and 11%, 16% and 9% in roots at all three time points, respectively (*P* < 0.05). Thus, the roots of strawberry were earlier subjected to membrane injuries than leaves, but quickly protected by ALA from the damages compared with the leaves.

Furthermore, the free proline content was increased in both leaves and roots of strawberry after PEG treatment (Fig. 2K-L). ALA co-addition significantly improved proline accumulation in the roots (*P* < 0.05) but tended to depress the accumulation in the leaves although the effect was not all significant at *P* = 0.05. These suggest that free proline is a complex index for plant water stress.

### Transcriptomic analysis for the strawberries under PEG and ALA treatments

**Fig. 3:**
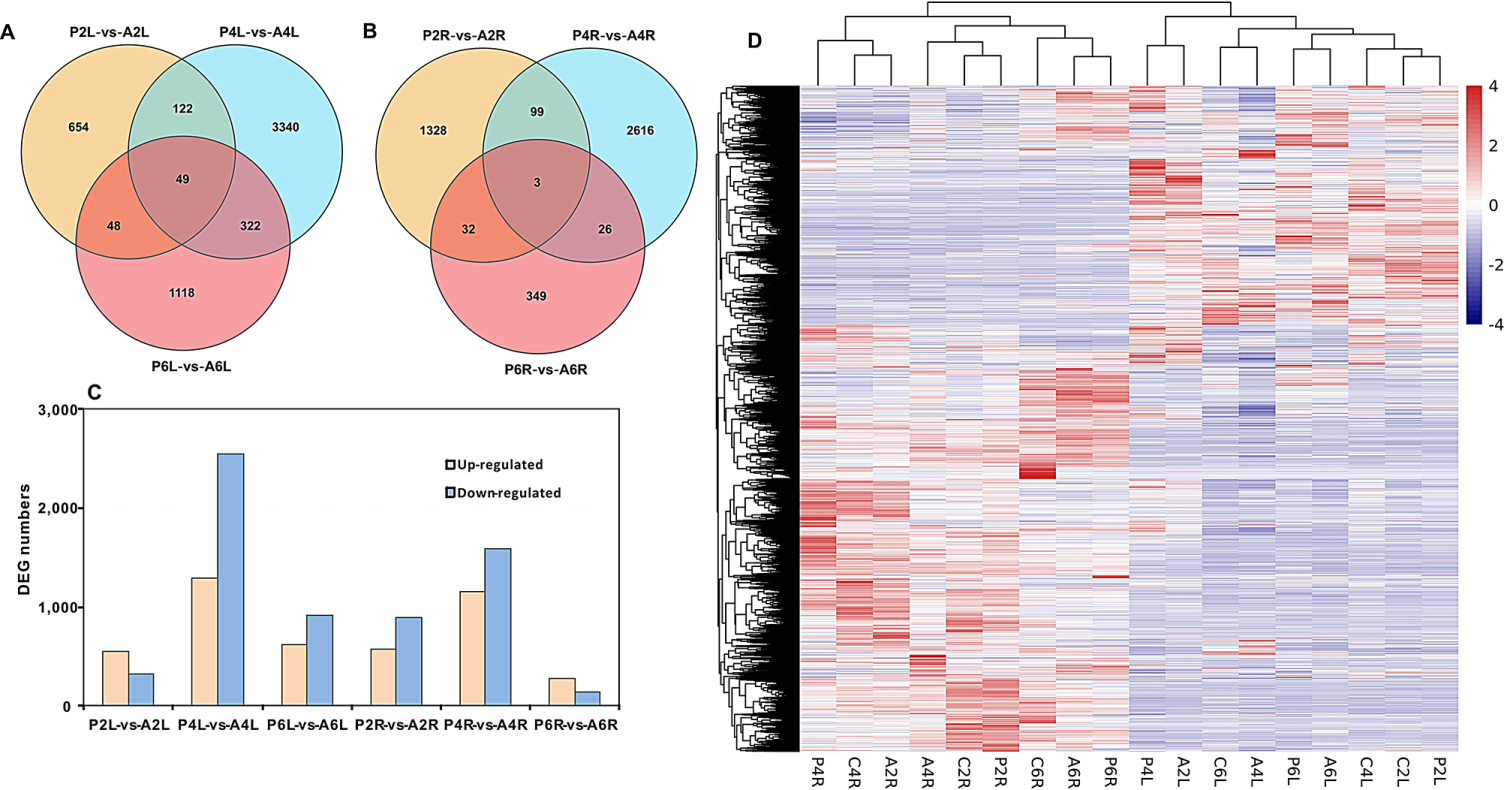
The differentially expressed genes (DEGs) between PEG and PEG + ALA treatments in leaves and roots. Veen diagrams depict the shared and unique DEG numbers among the different treatments in leaves (**A**) and roots (**B**); **C**: the numbers of up-regulated and down-regulated DEGs between different treatments; **D**: the expression heatmap of DEGs under different treatments in leaves and roots. The color-bar represents the expression quantities after standardization by the pheatmap package (https://cran.r-project.org/web/packages/pheatmap/) of R script. The genes and treatments were under clustering analysis by R script. The C2L, C4L and C6L represent the leaves of control plantsat the 2nd, 4th, and 6th day, respectively, and the C2R, C4R and C6R represent the roots of control plantsat the 2nd, 4th, and 6th day. The P2L, P4L and P6L represent the leaves of PEG treated plants at the 2nd, 4th, and 6th day, respectively, and the P2R, P4R and P6R represent the roots of PEG treated plants at the 2nd, 4th, and 6th day. Accordingly, A2L, A4L, A6L, A2R, A4R and A6R represent the leaves and roots of PEG + ALA treated plants at the 2nd, 4th, and 6th day, respectively. In all comparisons, genes from the PEG treatment were set as the controls

Transcriptomes of leaves and roots were determined to uncover molecular mechanisms of ALA-applied strawberries suffering from osmotic stress, which were harvested from strawberry plants under control, PEG, and PEG + ALA treatments for two, four and six days with three replications. There were over 2.4 billion paired-end raw reads among all the 54 samples, in which approximately 99.7% of them were kept as clean reads after filtering out the adapter and low-quality reads (Table S2). The clean reads were unique mapped to the reference genome *F. x ananassa* ‘Camarosa’ v1.0a1 (Table S3) for read counts and FPKM calculation. Subsequently, genes that were differentially expressed (DEGs) between PEG and PEG + ALA treatments in the leaves and roots for 2, 4 and 6 days were identified, respectively. A total of 9315 DEGs were identified between the different treatments at the same time and same tissue. Ten DEGs were randomly selected for RT-qPCR validation, showing that pearson’s correlation coefficients of RT-qPCR and RNA-Seq results were mostly higher than 0.7 (Table S4), which supported the reliability of the RNA-Seq data. In the treated-leaves, 873, 3833, and 1537 DEGs were detected in the comparisons of P2L (the leaf sample from PEG-treated plants for 2 days) vs. A2L (the leaf sample from PEG + ALA treated plants for 2 days), P4L (the leaf sample from PEG-treated plants for 4 days) vs. A4L (the leaf sample from PEG + ALA treated plants for 4 days), and P6L (the leaf sample from PEG-treated plants for 6 days) vs. A6L (the leaf sample from PEG + ALA treated plants for 6 days), respectively (Fig. 3A). In the roots, less DEG numbers of 1462, 2744, and 410 were found in the P2R (the root sample from PEG-treated plants for 2 days) vs. A2R (the root sample from PEG + ALA treated plants for 2 days), P4R (the root sample from PEG-treated plants for 4 days) vs. A4R (the root sample from PEG + ALA treated plants for 4 days) and P6R (the root sample from PEG-treated plants for 6 days) vs. A6R (the root sample from PEG + ALA treated plants for 6 days), respectively (Fig. 3B). It is clear that more DEGs were stimulated in roots by PEG and ALA application at the second day than those in leaves, indicating that exogenous ALA can rapidly help roots to confront osmotic stress at the early stage than the leaves. Furthermore, DEGs from the two tissues were all heavily stimulated at four days and relatively weakly induced at six days, respectively. Overall, more DEGs in leaves than in roots imply that more leaf genes exhibited strong responses to ALA than those of roots under PEG stress. Low numbers of shared genes between these comparison groups manifest that different DEGs responded to the ALA treatment at different periods in PEG-treated strawberry leaves and roots. Except P2L vs. A2L and P6R vs. A6R, the DEGs of the other four comparisons were more down-regulated rather than up-regulated by the exogenous ALA (Fig. 3C). The transcript abundance of all DEGs were plotted as the heatmap (Fig. 3D), in which most of them showed tissue-specific expression pattern among the three treatments for all time points.

### GO and KEGG enrichment of DEGs in PEG-stressed strawberries after ALA treatment

**Fig. 4:**
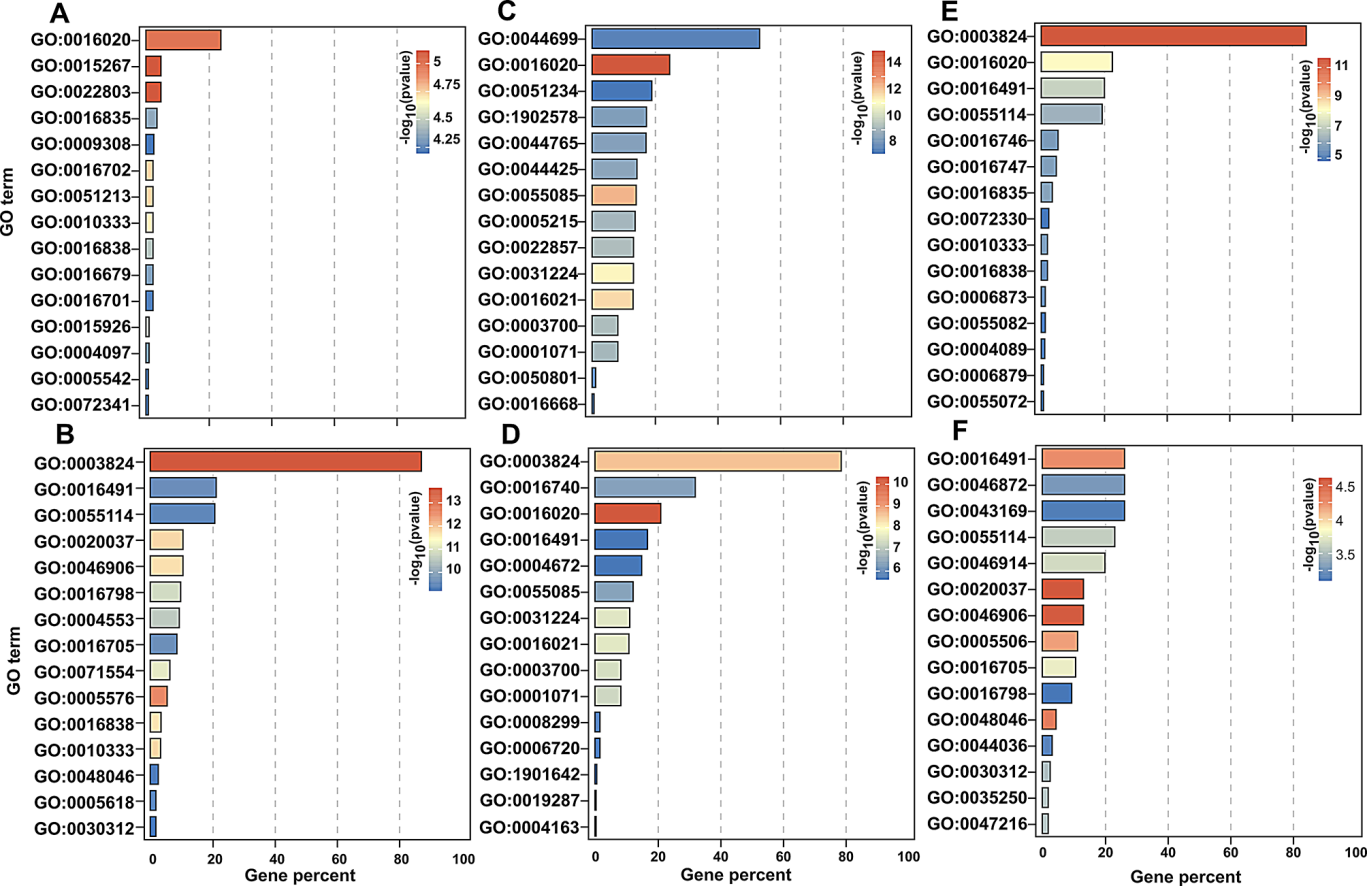
The GO enrichment analysis of the DEGs between PEG and PEG + ALA treatments in leaves and roots. **A**: P2L-vs-A2L; **B**: P2R-vs-A2R; **C**: P4L-vs-A4L; **D**: P4R-vs-A4R, **E**: P6L-vs-A6L; **F**: P6R-vs-A6R. The description of GO terms is listed in Table S5

**Fig. 5:**
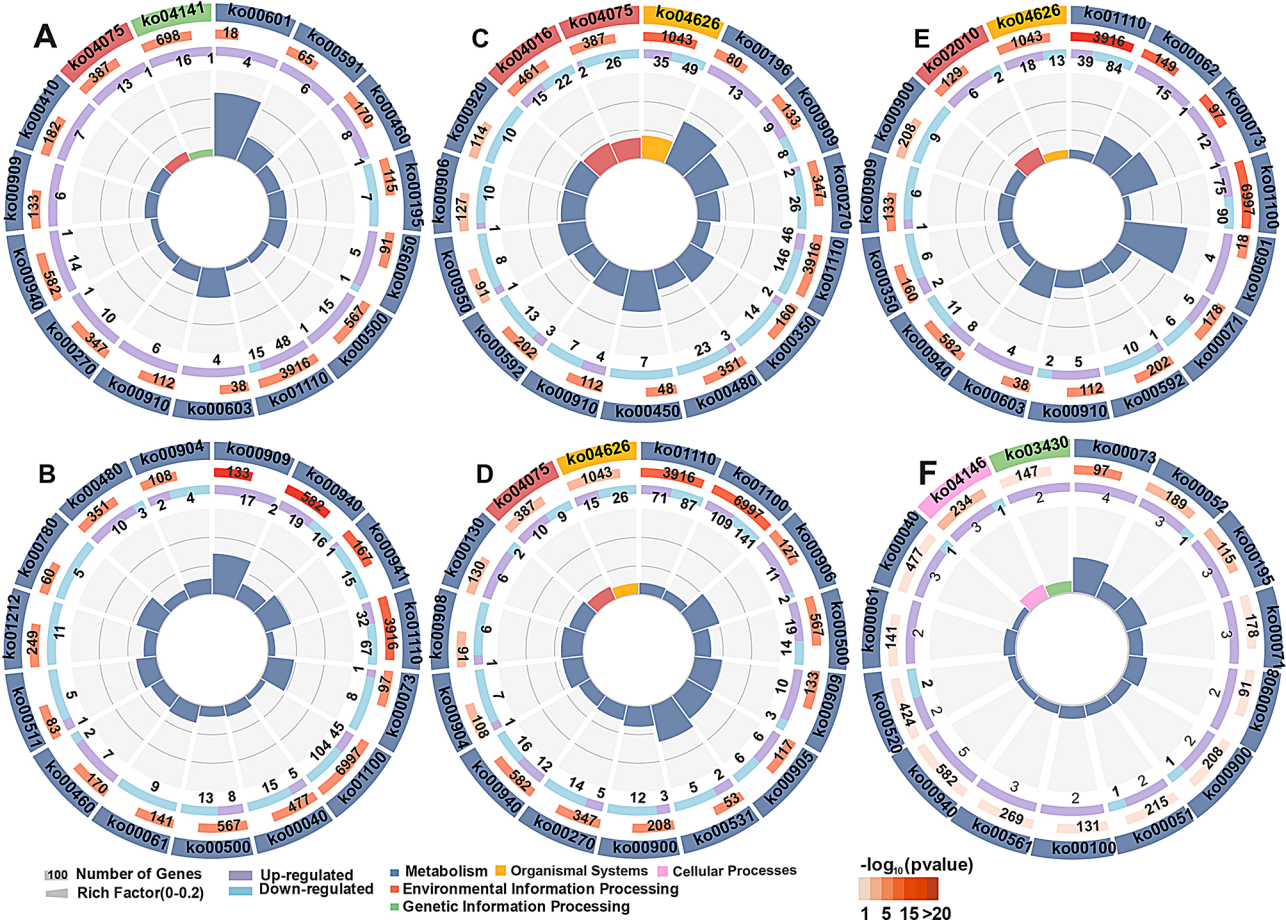
The KEGG enrichment analysis of the DEGs between PEG and PEG + ALA treatments in leaves and roots. **A**: P2L-vs-A2L; **B**: P2R-vs-A2R; **C**: P4L-vs-A4L; **D**: P4R-vs-A4R, **E**: P6L-vs-A6L; **F**: P6R-vs-A6R. The description of KEGG pathways is listed in Table S6

To explore the regulatory functions of ALA on strawberries under PEG stress, the GO (Fig. 4 and Table S5) and KEGG enrichment analysis (Fig. 5 and Table S6) were carried out among the DEGs in leaves and roots. Because of diverse genes were differentially expressed across the three time points, the GO terms and KEGG pathways were distinct both between the different tissues and between the different stages. In the case of GO enrichment, a few DEGs in leaves at the second day were significantly clustered in ‘membrane’ (GO:0016020), ‘channel activity’ (GO:0015267), ‘passive transmembrane transporter activity’ (GO:0022803), as well as ‘oxidoreductase activity’ (GO:0016702). These suggest that a small subset of genes involving cellular component and molecular function were stimulated by ALA treatment in response to osmotic stress at the early stage. However, at the same period, the GO cluster of ‘catalytic activity’ (GO:0003824), ‘oxidoreductase activity’ (GO:0016491), ‘oxidation-reduction process’ (GO:0055114) and ‘cell wall organization or biogenesis’ (GO:0071554) possessed much more DEGs from roots. These indicate that the root genes were more widely and rapidly convened by exogenous ALA to recover the physiological and biochemical responses in cells as well as the cell barriers underlying the PEG stress. It is interesting to see that 140 DEGs were clustered in ‘heme binding’ (GO:0020037) and ‘tetrapyrrole binding’ (GO:0046906) classes. These indicate that ALA, as the precursor of tetrapyrroles, quickly regulates its downstream reactions in strawberry roots subjected to osmotic stress. As the treatment time was up to four days, a lot of leaf DEGs enriched in the GO classes with respect to stress tolerance, such as ‘transmembrane transport’ (GO:0055085), ‘transcription factor activity’ (GO:0003700) and ‘oxidoreductase activity’ (GO:0016491) and so on. Besides, there was a term named as ‘ion homeostasis’ (GO:0050801) gathering, which demonstrates that these genes can be recruited by ALA to maintain ion balance in PEG-treated leaf cells. ALA also regulated genes involved in isoprenoid biosynthesis and metabolism (GO:0008299 and GO:0006720) in roots at the fourth day. They are important secondary metabolites in response to various stresses [52]. At the sixth day, although less DEGs clustering in GO terms both appeared in leaves and roots, a series of drought-resistant response, including oxidation-reduction process (GO:0016491 and GO: 0055114), intracellular homeostasis (GO:0006873 and GO:0055082), and so on, were persistently stimulated by exogenous ALA for osmotic tolerance.

In the aspect of KEGG enrichment, all DEGs from the six comparison groups preferred gathering in the KO class of Metabolism, suggesting that ALA is involved in an extensive range of metabolic regulation in the strawberry leaves and roots in response to PEG stress, containing ‘Biosynthesis of secondary metabolites’ (ko01110), ‘Starch and sucrose metabolism’ (ko00500), ‘Photosynthesis’ (ko00195), ‘Sesquiterpenoid and triterpenoid biosynthesis’ (ko00909), and so forth (Fig. 5 and Table S6). Furthermore, the root DEGs significantly clustered in ‘Glutathione metabolism’ (ko00480) and ‘Diterpenoid biosynthesis’ (ko00904) also sensitively responded to ALA for osmotic stress tolerance at early stage. However, at this stage, some leaf-specific pathways such as ‘Plant hormone signal transduction’ (ko04075), ‘Linoleic acid metabolism’ (ko00591) and ‘Photosynthesis’ (ko00195), and leaf-root-shared pathway of ‘Biosynthesis of secondary metabolites’ (ko01110) were also enriched, in which the ‘Plant hormone signal transduction’ pathway was also gathered in leaves and roots after four days treatment, and the ‘Biosynthesis of secondary metabolites’ were simultaneously enriched in the two tissues across the PEG and ALA treatments, except in roots at the sixth day. It is noteworthy that part of the DEGs in ‘Plant hormone signal transduction’, ‘Linoleic acid metabolism’ and ‘Biosynthesis of secondary metabolites’, comprising *MYC2*, *GH3.5*, *AOS1*, *AOC*, *OPR3*, *JMT* and so on, were involved in the JAs biosynthesis, catabolism and signaling (Table S6). These imply that JAs might be an important factor in the ALA-mediated osmotic tolerance pathway in strawberries.

### Temporal expression trends of DEGs in PEG-stressed strawberries after ALA treatment

**Fig. 6:**
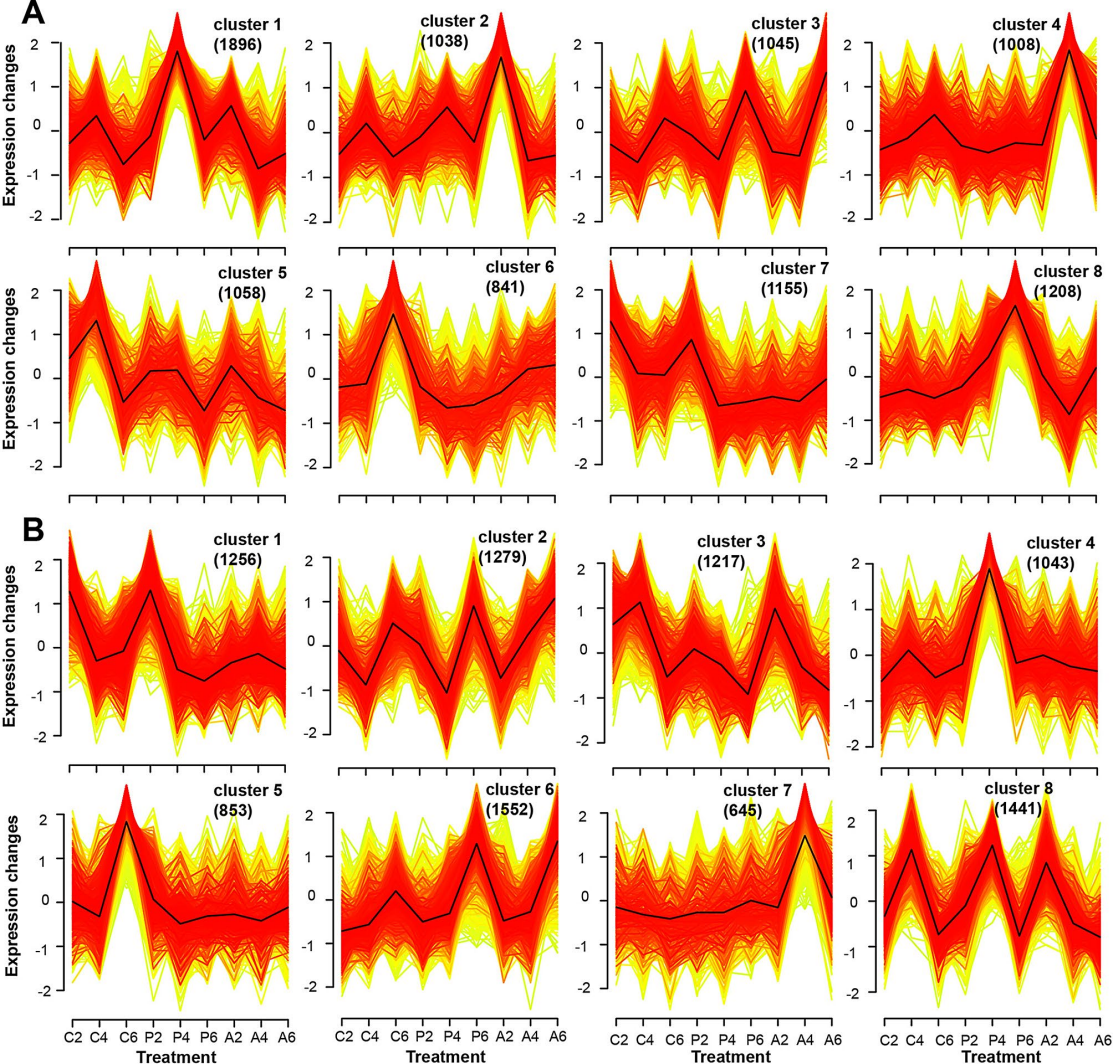
The expression patterns of DEGs under different treatments in leaves **(A)** and roots **(B).** The red lines mean gene expression with higher membership values, followed by the yellow ones. The numbers in brackets mean the DEG numbers in each cluster. The cluster centers are marked as the black lines

To further understand the expression profile of all DEGs under different treatments, Mfuzz was used to perform the temporal expression patterns and classify them into eight expression clusters in strawberry leaves and roots, respectively (Fig. 6). As far as the expression trends of leaf DEGs was concerned, cluster 1 and 6 owned the highest and lowest genes among the clusters, respectively, while the other six ones had similar gene numbers around 1000 (Fig. 6A). The expression levels of DEGs from cluster 1 and 8 showed up-regulation after PEG treatment, but down-regulation after ALA addition. DEG expression levels from cluster 2 and 3 were gradually induced by PEG and PEG + ALA, especially those of cluster 2 were rapidly enhanced by ALA at the early stage. Different from these four clusters, DEG expression of cluster 4, 5, 6, and 7 was decreased by the PEG-stimulated osmotic stress in strawberry leaves. When exogenous ALA was treated with PEG, three distinctly expressed trends were detected among them, that is, DEGs up-regulation in cluster 4 and 6, no obvious change in cluster 5, and down-regulation in cluster 7. Therefore, the DEGs from cluster 2, 3, 4 and 6 were positively responsive to ALA, especially containing the strongly positive response in cluster 2 and 4 at early and middle stages, respectively; but those of cluster 1, 7 and 8 negatively responded to ALA in strawberry leaves. Interestingly, over half of the DEGs involved in JAs biosynthesis, catabolism, and signaling were predominantly located in clusters 2 and 4, exhibiting relatively high membership values. This suggests their strong and positive response to ALA in strawberry leaves under osmotic stress (Table S7 and S8).

Moreover, different expression profiles were also revealed in the DEGs of strawberry roots (Fig. 6B). The minimum DEGs were classified into cluster 7 followed by cluster 5, and the maximum genes were detected in cluster 6. The PEG only treatment caused DEG expressions with no clear variation in cluster 1, 2, 7 and 8, but PEG + ALA treatment led to down-regulation in cluster 1, up-regulation in cluster 7, and remaining no change in cluster 2 and 8. These demonstrate that DEGs of cluster 1 and 7 were negative and positive response to ALA application, respectively, in which the ALA played the dominant roles than PEG. DEGs from cluster 4 and 6 showed obviously increased expression levels after PEG treatment, while those of PEG + ALA were visibly inhibited in cluster 4, and no marked change in cluster 6. The results indicate that the DEGs from cluster 4 exhibited strong response to osmotic stress, which can be extinguished by ALA in strawberry roots. Although the remaining cluster 3 and 5 both showed decreasing expression trends between control and PEG treatments, only cluster 3 was positive response to ALA and its expression was recovered to the control levels in roots. It is noteworthy that approximately half of the JA-related DEGs congregated in clusters 3 and 7, further illustrating that JAs may play significant and beneficial roles in ALA-stimulated osmotic tolerance in strawberry roots (Table S8). Therefore, the expression profiles of the DEGs related to JA biosynthesis, catabolism and signaling might reveal the key response mechanism of osmotic stress enhancement by exogenous ALA.

### Expression patterns of the DEGs related to jasmonates

**Fig. 7:**
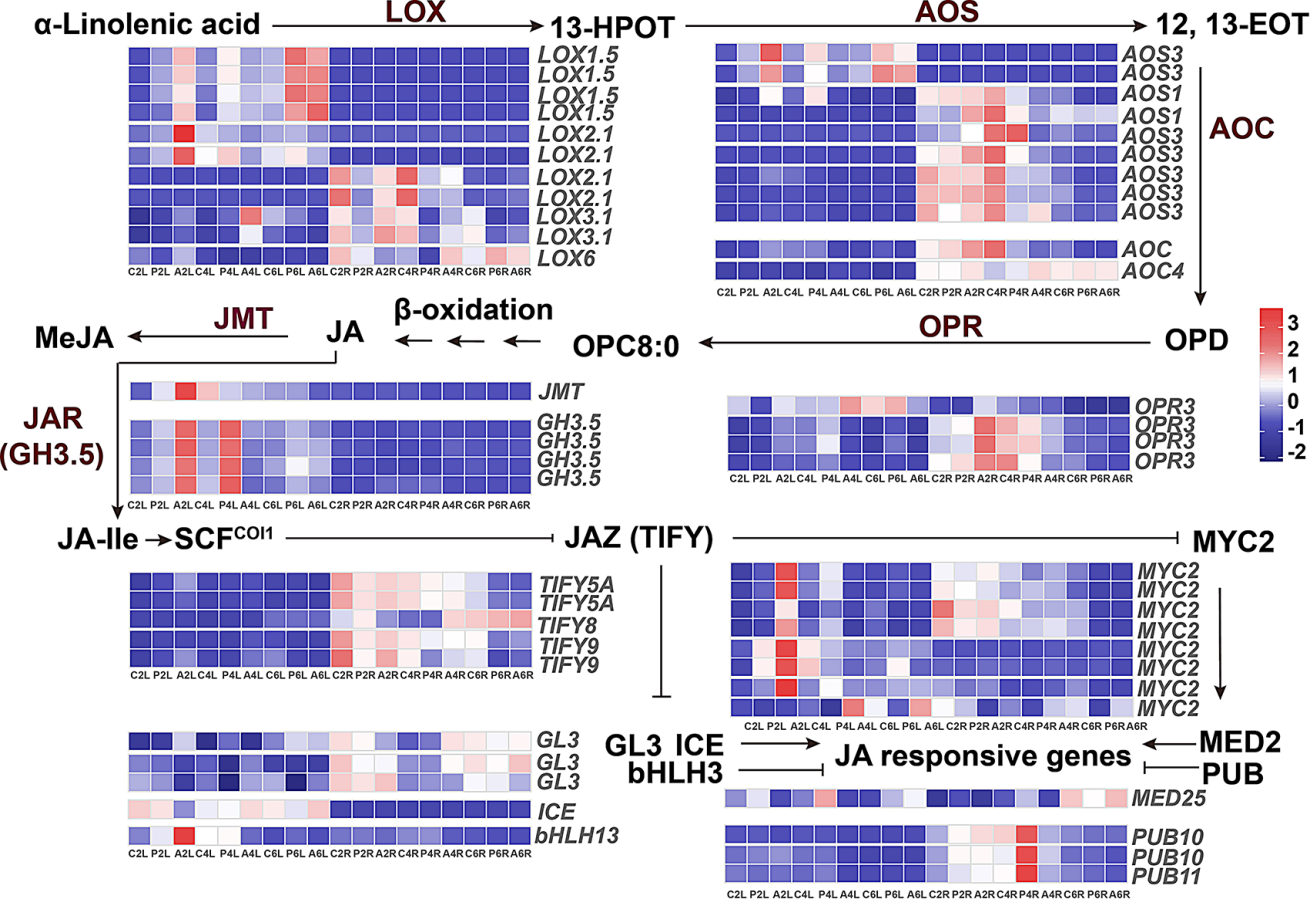
The expression heatmap of DEGs related to JAs biosynthesis, catabolism and signaling. The order of the small squares is C2L, P2L, A2L, C4L, P4L, A4L, C6L, P6L, A6L, C2R, P2R, A2R, C4R, P4R, A4R, C6R, P6R, and A6R. JA biosynthesis is exhibited through the octadecanoid pathway here. 13-HPOT: (13*S*)-hy- droperoxyoctadecatrienoic acid; 12, 13-EOT: 12, 13(S)-epoxyoctadecatrienoic acid; OPDA: 12-oxophytodienoic acid; OPC8:0: 3 − 2(2‘(Z)-pentenyl) cyclopentane-1-octanoic acid; COI1: F-box coronatine-insensitive protein 1; SCF^COI1^: Skp/Cullin/F-box CORONATINE INSENSITIVE1 complex; JAZ: Jasmonate ZIM-domain protein; LOX: lipoxygenase; AOS: allene oxide synthase; AOC: allene oxide cyclase; OPR: OPDA reductase; JMT: JA methyl transferase; JAR/GH3.5: JA conjugate synthetase/indole-3-acetic acid-amido synthetase; MED25: mediator of RNA polymerase II transcription subunit 25; PUB: U-box domain-containing protein; GL3: transcription factor GLABRA 3; basic-helix-loop-helix (bHLH) transcription factor MYC2, ICE and bHLH

The expression heatmaps of DEGs involved in JAs biosynthesis, catabolism and signaling pathways were exhibited to uncover the regulation effect of exogenous ALA on them at different stages after osmotic stress. Most of them presented tissue-specific expression profiles across all treatment times, indicating that paralogous genes are assigned to the different tissues for carrying out the similar function in strawberries (Fig. 7). Part of *lipoxygenase* (*LOX*s), *allene oxide synthase* (*AOS*s) and *allene oxide cyclase* genes (*AOC*s), as the upstream genes in JA biosynthesis pathway, strongly responded to ALA treatment at the early stage in strawberry leaves, particularly the *LOX2.1*, *AOS3* and *AOC* from leaf expression cluster 2 (Table S8). However, most of them cannot be positively stimulated by ALA in leaves at the later period and in roots for all the time points. On the other hand, some of the *LOX*s, *AOS*s and *AOC*s revealed root-specific expression profile, which were also in favor of responding to ALA application at the second and fourth instead of sixth day. These findings suggest that ALA can stimulate the tissue-specific genes for quickly switching on JA biosynthesis in strawberry plants under PEG-induced osmotic stress. Subsequently, different *12-oxophytodienoic acid reductase 3* (*OPR3*) genes were positively induced by ALA in roots at early stage, but not at other stages. The effect of ALA on *OPR3* from leaf cluster 4 can be enhanced in strawberry leaves at the fourth day, but not at other stages. Similarly, *JA methyl transferase* (*JMT*) and *JA conjugate synthetase/indole-3-acetic acid-amido synthetase* (*JAR*/*GH3.5*) were also conspicuously responsive to ALA treatment in leaves at the second day, suggesting that ALA promoted JA biosynthesis at the early stage when strawberry was stressed by PEG. Since JAR is responsible for JA-Ile synthesis, which is the most active JA, the upregulated *JAR* might facilitate to improve osmotic stress tolerance of strawberries as quickly as possible. In the JA signaling pathway, the *Jasmonate ZIM-domain* (*JAZ* /*TIFY*) DEGs had higher different expression in roots than leaves, four of them (two *TIFY5A* and two *TIFY9*) in roots were enhanced by ALA at the early stage, compared with the PEG only, and the remaining one (*TIFY8*) was activated at the later stage. As we know, JA-Ile improves the co-receptor of SCF^COI1^-JAZ formation for releasing the inhibition of JAZ on the downstream genes [53], such as *MYC2*s, *mediator of RNA polymerase II transcription subunit 25* (*MED25*s), *GLABRA 3* (*GL3*s), and *basic-helix-loop-helix* (*bHLH*s). The most noticeable downstream member is *MYC2s*, playing important roles in JA signaling. Five of them were differentially expressed and positively responsive to ALA in strawberry leaves at the second day, suggesting that the JA-JAZ-MYC2 signaling switch might be very sensitive to ALA treatment under osmotic stress.

### WGCNA of the differentially expressed genes in strawberries

**Fig. 8:**
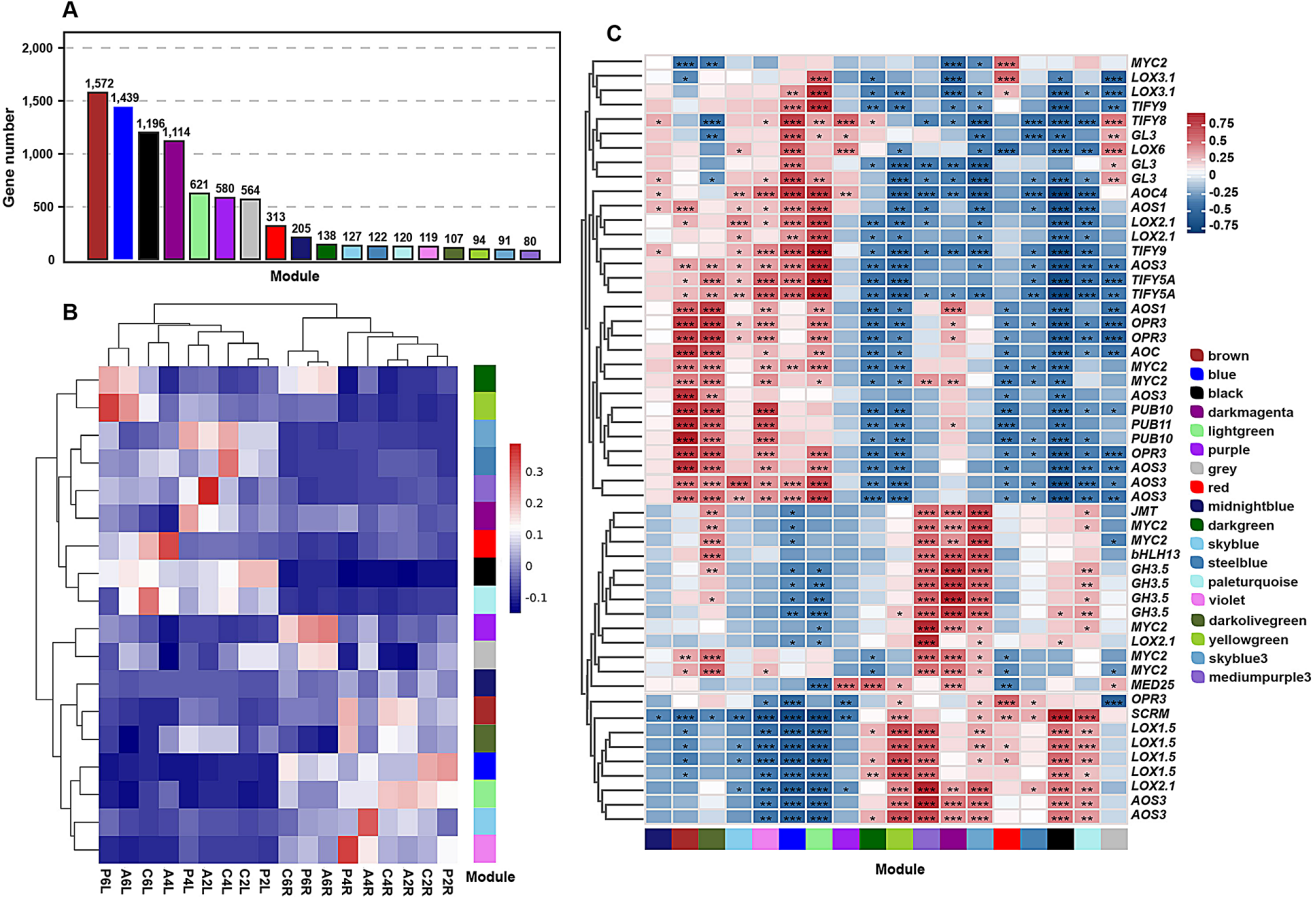
The module segmentation and characteristics based on WGCNA. **A**: The gene numbers of each module according to WGCNA; the colors mean the module ID. **B**: The average expression quantities of the modules in each treatment; the bar with numbers represents the expression quantities after standardization by R script. The modules and treatments were under clustering analysis by R script. **C**: The correlation between modules and the JA-related gene expressions; one, two and three asterisks (*, ** and ***) mean the significance under *P* < 0.05, *P* < 0.01 and *P* < 0.001, respectively; the bar with numbers represents the correlation indexes

**Fig. 9:**
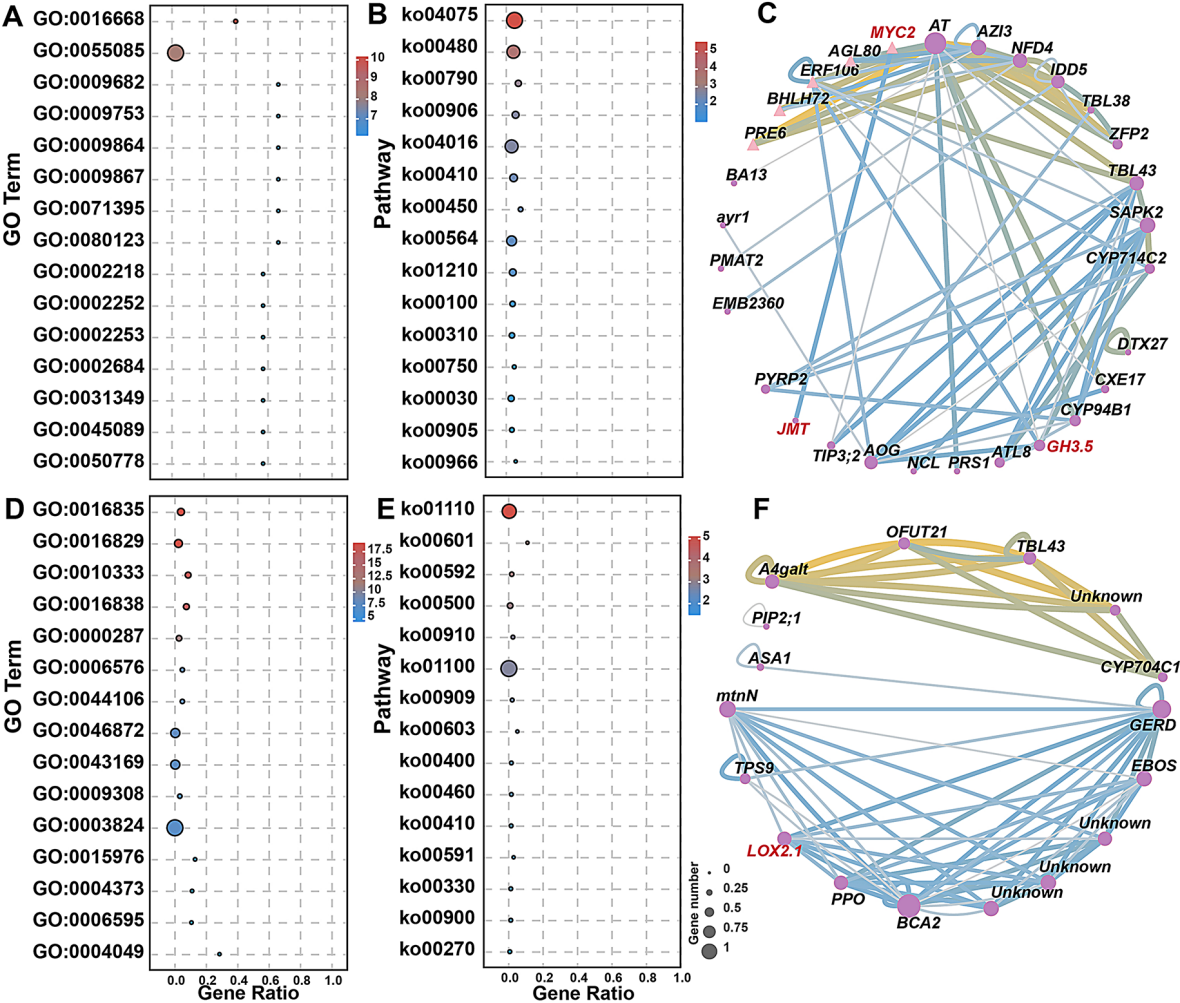
The characteristic of DEGs in darkmagenta **(A-C)** and mediumpurple3 **(D-F)** modules. **A** & **D**: The GO enrichment analysis of all genes in dark magenta and mediumpurple3 modules, respectively. The description of GO terms is listed in Table S9. B & E: The KEGG enrichment analysis of all genes in dark magenta and mediumpurple3 modules, respectively. The description of KEGG pathways is listed in Table S10. The color bars are -log_10_(p value). **C** & **F**: Co-expression network of genes with high connectivity (top 100) in dark magenta and mediumpurple3 modules, respectively. The pink triangles mean transcription factors, and the circle nodes mean non-transcription factors. The larger the node sizes, the higher the connectivity. The lines become thicker as the weight values increase. The yellow lines mean higher weight values, followed by the blue and grey ones. The JA-related genes are marked as red color. Unknown mean genes with unknown function so far, and the gene ids are maker-Fvb6-2-snap-gene-177.38, snap_masked-Fvb3-3-processed-gene-7.20, maker-Fvb6-4-augustus- gene-62.31, and maker-Fvb6-4-snap-gene-62.49. *A4galt*: lactosylceramide 4-alpha-galactosyltransferase; *BCA*: carbonic anhydrase; *AGL80*: MADS-box protein *AGL*; *AOG*: glucosyltransferase abscisate beta-glucosyl- transferase-like; *ASA1*: anthranilate synthase alpha subunit 1; *AT*: acyl-transferase; *ATL8*: RING/U-box superfamily protein; *ayr1*: NADPH-dependent 1-acyldihydroxyacetone phosphate reductase; *AZI3*: azelaic acid induced 3; *BA13*: cytochrome P450 85 A; *CXE17*: carboxyesterase 17; *CYP*: cytochrome P450; *DXT27*: detoxification 27; *EBOS*: tricyclene synthase *EBOS*; *EMB2360*: glutathione reductase; *ERF106*: ethylene-responsive transcription factor ERF106; *GERD*: (-)-germacrene D synthase; *IDD5*: indeterminate(ID)-domain 5; *mtnN*: bark storage protein A-like; *NCL*: sodium/calcium exchanger; *NFD4*: nuclear fusion defective 4; *OFUT21*: rhamnogalacturonan I rhamnosyltransferase; *PIP2*: aquaporin; *PMAT2*: phenolic glucoside malonyltransferase 2-like; *PPO*: polyphenol oxidase; *PRE6*: transcription factor PRE6; *PRS1*: ribose-phosphate pyrophosphokinase 1; *PYRP2*: 5-amino-6-(5-phospho-D-ribitylamino)uracil phosphatase; *SAPK2*: serine/threonine-protein kinase; *TBL43*: richome birefringence like 43, O-acetyltransferase activity; *TIP3-2*: aquaporin-like; *TPS9*: terpene synthase 9; *ZFP2*: zinc finger protein 2

For further discovering the key JA-related DEGs response to ALA under PEG treatment, WGCNA was performed to identify 18 modules, in which the DEGs distribution were extremely uneven among them (Fig. 8A). For example, over 1000 DEGs were clustered in the brown, blue, black, and dark magenta modules, while yellow green, skyblue3 and medium purple3 modules possessed less than 100 DEGs, respectively. The tissue-specific expression pattern was still demonstrated by the average expression quantities of the modules in each treatment, indicating that DGEs of dark green, yellow green, skyblue3, steel blue, mediumpurple3, dark magenta, red, black, and pale turquoise modules had signally higher expression levels in leaves than those in roots, whereas the opposite phenomenon appeared in the other nine modules (Fig. 8B). In the leaf-specific expressed modules, the DEGs from dark magenta and medium purple3 were positively responsive to ALA treatment at early stage. Quite interestingly, some JA-related GO and KO terms were significantly enriched in these two modules (Fig. 9, Table S9 and 10), containing GO terms of ‘response to jasmonic acid’ (GO:0009753), ‘induced systemic resistance’ (GO:0009682 and GO:0009864), ‘jasmonic acid mediated signaling pathway’ (GO:0009867) and ‘cellular response to jasmonic acid stimulus’ (GO:0071395); as well as the KO pathways of ‘Plant hormone signal transduction’ (ko04075) and ‘MAPK signaling pathway-plant’ (ko04016), ‘Biosynthesis of secondary metabolites’ (ko01110), ‘alpha-Linolenic acid metabolism’ (ko00592), ‘Metabolic pathways’ (ko01100) and ‘Linoleic acid metabolism’ (ko00591). Similarly, GO and KEGG enrichment involved in JA were also detected in the two root-specific modules, brown and light green, which were positively stimulated by exogenous ALA at the early stage likewise (Fig. 8B, Table S9 and S10). Moreover, the correlation between of JA-related gene expressions and all modules displayed that more JA-related DEGs had correlation indexes > 0.8 with *P* < 0.001 in the brown, light green, dark magenta and medium purple3 modules (Fig. 8C and Table S11). In other words, it was deeply discovered and confirmed that the four modules quickly respond to exogenous ALA and were more associated with JA biosynthesis, catabolism and signaling than the others.

In addition, the weight values between DEGs in the four modules were also calculated for constructing co-expression networks and discovering hub genes, respectively. Regrettably, the JA-related DEGs from brown and light green modules had lower weights among all gene pairs, showing their less vital roles in their own module to answer exogenous ALA in strawberry roots subjected to osmotic stress. Nevertheless, relatively higher weight values (top 100) were detected in JA-related DEGs from dark magenta and medium purple3 modules, representing that these genes were identified as members of hub genes and relatively crucial to ALA-induced osmotic tolerance in strawberry leaves. According to the results above, the visualization of GO, KEGG enrichment and co-expression networks was only executed in darkmagenta and mediumpurple3 modules (Fig. 9). On the one hand, *GH3.5* had higher connectivity with other genes in the aspect of darkmagenta module, in especial with *SAPK2*/*SnRK2* (serine/ threonine-protein kinase); and the *JMT* co-expressed with *MYC2* independent of other genes. On the other hand, reliable co-expressions between *LOX2.1* and *GERD* (germacrene D synthase), *EBOS* (tricyclene synthase/(E)-beta-ocimene synthase) and *PPO* (polyphenol oxidase) were also explored in mediumpurple3 module. Therefore, these findings illustrate that ALA stimulate a wide range of networks, which directly or indirectly participate the JA regulatory pathways for strawberries osmotic tolerance.

### Effect of exogenous ALA treatment on the JAs content in strawberry under PEG stress

**Fig. 10:**
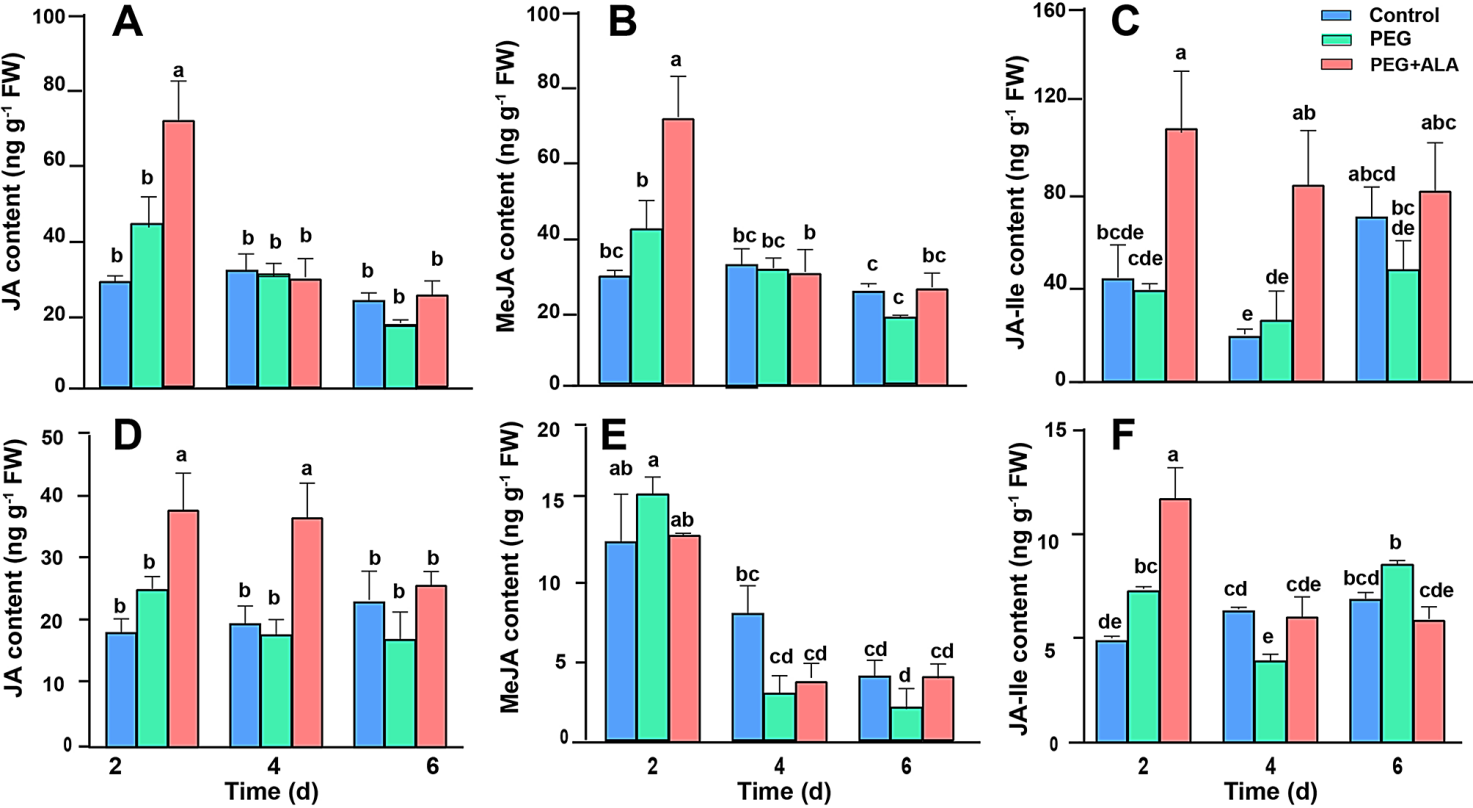
Effect of ALA treatment on the JAs content in the leaves (**A**-**C**) and roots (**D**-**F**) of strawberry after PEG stress for different days. The same lowercases in each panel represent no significant difference at *P* = 0.05 level

The content of JA, MeJA, and JA-Ile in the leaves and roots of strawberry was measured by HPLC-MS (Fig. 10). The results showed that PEG stress did not greatly affect the JAs content, but additional ALA application significantly induced JA, MeJA, and JA-Ile increases in the strawberry stressed by PEG. In the leaves, the increases of JA and MeJA induced by ALA was observed after 2-day treatment, while the significant increase of JA-Ile was found in the 2- and 4- day treatment. In the roots, the effect of JA increase was found in the 2- and 4-day treatment while JA-Ile was significantly increased in the 2-day treatment when compared with PEG stress only. Nevertheless, we did not observe any difference of MeJA content in the roots of strawberry between treatments. This may suggest that JA biosynthesis and metabolism is not synchronized in different tissues. Generally, JAs were not sensitive to PEG stress, while ALA induced rapid accumulation of JAs in strawberry when plants were stressed by PEG.

### Effect of exogenous MeJA and the inhibitor on the osmotic tolerance of strawberries

**Fig. 11:**
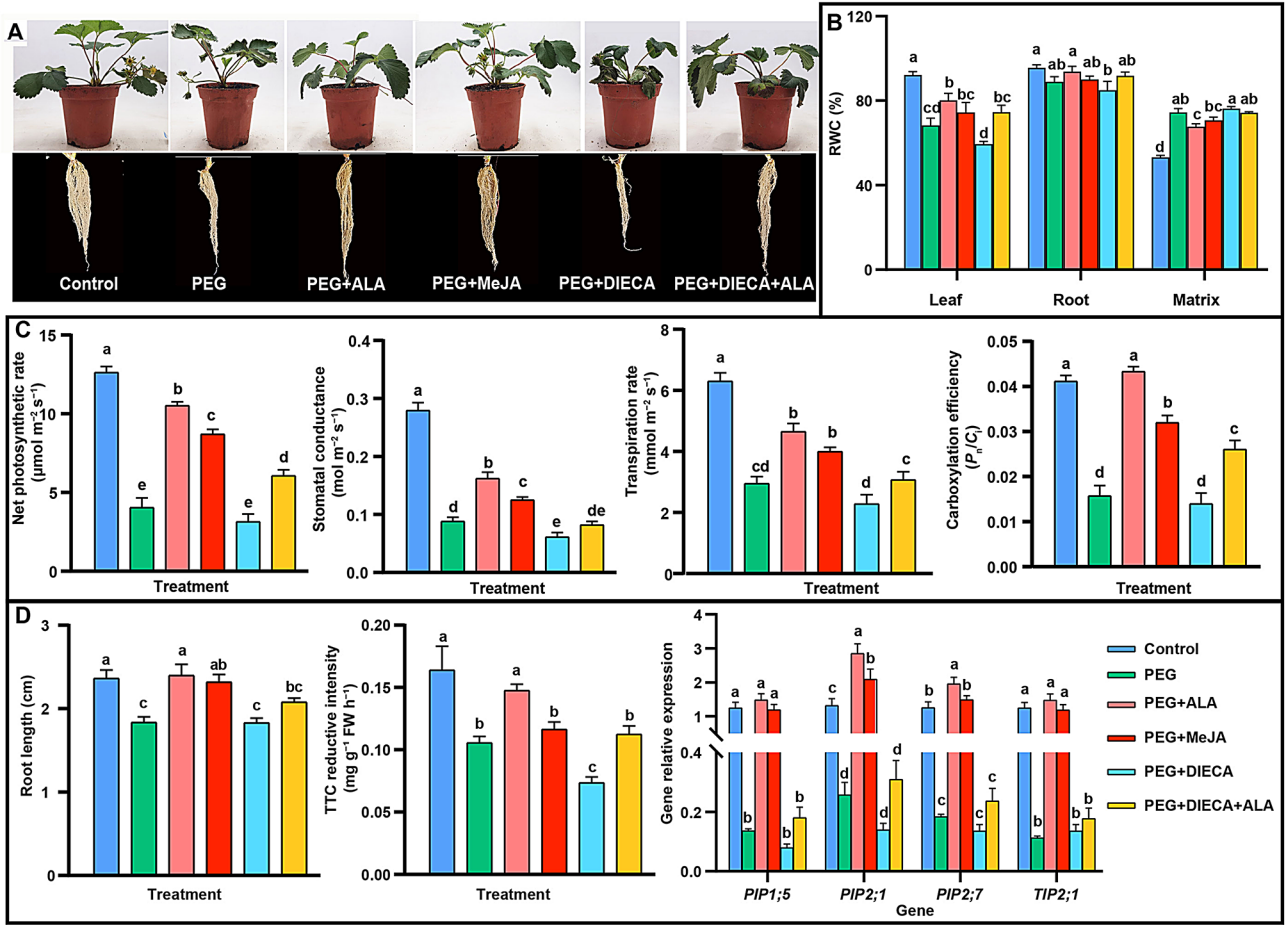
The effects of ALA and MeJA on osmotic tolerance of strawberries for six days. **A**: The phenotypes of strawberries under different treatments. **B**: The RWC in strawberry leaves, roots, and the matrix. **C**: The gas exchange parameters of strawberry leaves. **D**: The root lengths, TTC reductive intensity and expressions of some aquaporin genes. The same lowercases in each index or gene represent no significant difference at *P* = 0.05 level

Based on analyses above, we deduced that JA might be involved in ALA-promoted strawberry drought tolerance. Therefore, a new series of experiments were performed with exogenous MeJA and its biosynthetic inhibitor DIECA applied to strawberries for six days (Fig. 11). The results showed that application of MeJA can relieve the drought damages both on leaves and roots brought by PEG stress, an effect similar with ALA, while addition of DIECA aggravated the drought injuries, which can be alleviated by additional application of ALA. When the water status was analyzed, the RWC in the leaves of PEG stressed plants was significantly decreased while ALA prevented the decreasing. MeJA had similar alleviating effect, although the difference was not significant at *P =* 0.05. Nevertheless, DIECA dramatically depressed the RWC, which can be reversed by additional application of ALA (*P* < 0.05). A similar trend of RWC changes can be found in the roots of strawberry in different treatments. Conversely, the RWC in the culture matrix stressed by PEG only was significantly higher than that of the control, while that in the ALA treatment was significantly lower than PEG stress only. These suggest that PEG stress inhibited root water absorption from the matrix while ALA promoted water absorption by roots. Similar effects can be observed in other treatments. When MeJA was added, the matrix RWC was lower than PEG stress only, although *P* > 0.05; when DIECA was added, the RWC was significantly higher than PEG + MeJA (*P* < 0.05), which was decreased by additional ALA treatment. The findings are consistent with Fig. 1, and infer that JAs play indispensable role in ALA-mediated water absorption under osmotic stress.

In terms of the gas exchange parameters of leaves (Fig. 11C), PEG markedly decreased leaf *P*_n_, *G*_s_, *T*_r_ and *P*_n_/*C*_i_, while exogenous ALA or MeJA significantly alleviated the depression induced by PEG, for which ALA worked even better than MeJA (*P* < 0.05). These parameters were further depressed under PEG + DIECA, especially *G*_s_, which was significantly lower than PEG only (*P* < 0.05). When ALA was applied with PEG and DIECA, the *P*_n_, *T*_r_, and *P*_n_/*C*_i_ were significantly reversed, demonstrating that JAs partially participate in the ALA-enhanced photosynthetic capacity for stronger osmotic tolerance.

For the effects of ALA and MeJA on root systems, similar variations also appeared in root lengths and activity. PEG treatment led to inhibition of root growth and activity (*P* < 0.05), where the remissions of ALA were better than MeJA. It is worth noting that the root activity was decreased by 24% with DIECA added compared with PEG alone but improved by 41% after additional application of ALA. When the root activity of PEG + ALA was compared with that of PEG + ALA + DIECA, it was found that the latter was only 74% of the former. These suggest that inhibition of JA biosynthesis impaired the activity of strawberry roots under PEG stress, and ALA had a preventive effect against DIECA, nevertheless, the ALA-stimulated root activity was partially dependent on JA.

Furthermore, the expression levels of four aquaporin genes (including *PIP1;5*,* PIP2;1*,* PIP2.7*,* and TIP2;1*) in roots under different treatments also verified these phenomena. From Fig. 11D, PEG stress significantly decreased the gene expression, which can be reversed by addition of ALA or MeJA (*P* < 0.05). DIECA did not lead to distinctive down-regulation of the aquaporin genes compared with the PEG only, and addition of ALA up-regulated the expression of four genes to a certain extent. Nevertheless, the effect was not significant at *P* = 0.05. This suggests that ALA-induced aquaporin gene expression might be mainly dependent on JA signaling because inhibition of DIECA on aquaporin gene expression cannot be reversed by ALA.

## Discussion

ALA is a novel plant growth regulator that widely contributes to physiological and metabolic processes in plant growth, development, biotic and abiotic stress response [54]. Ever since ALA improving the plant drought tolerance was first discovered in barley [55] and wheat [56], this advantageous effect has been reported in an increasing number of plants, such as the maize [57], sunflower [10], Kentucky bluegrass [12], banana [58], and walnut [14]. Recently, the ALA-treated cultivated strawberry showed drought tolerance enhancement, which had obvious function to photosynthetic efficiency, root system and aquaporin gene expressions, thereby contributing to photosynthesis acceleration, biomass accumulation and water balance of plants subjected to osmotic stress [9]. In present study, ALA was still beneficial to photosynthesis gas exchanges, as well as the root growth and development of strawberries exposed to more serious osmotic stress (Figs. 1 and 20% PEG) than those in the previous study [9]. The osmotic stress led to lower RWC in strawberry leaves and roots, and higher RWC in matrix/substrate, while exogenous ALA can alleviate these injuries (Fig. 1). Our results indicate that ALA promotes the strawberry root to absorb more water from the substrate maintaining water balance under osmotic stress. What is more, only after treating with ALA for two days, the significantly accelerated antioxidant enzyme activities and the lower MDA accumulation were detected in roots (Fig. 2). Unlike the leaves, the roots were directly exposed to the osmotic stress, therefore the antioxidant system of strawberry roots was quickly responsive to ALA for improving their osmotic tolerance. These findings were consistent with the walnuts after treating with ALA under PEG-induced stress [14], manifesting that exogenous ALA might activate some mechanisms in plants for osmotic stress resistance. It is worth noticing that ALA gave rise to the tissue-specific response of H_2_O_2_ generation in PEG stressed strawberries, which was depressed in leaves, but promoted in roots (Fig. 2). These were consistent with the variation trend of H_2_O_2_ in leaves and roots from the ALA pretreated strawberries subjected to salt stress [59], which may be the ALA-induced adaptive strategies between different tissues to cope with the ecological stresses. In the leaf guard cells, H_2_O_2_ accumulation causes stomata closure, while the depression of H_2_O_2_ by ALA may be responsible for the increase of stomatal conductance (*G*_s_), intercellular CO_2_ concentration (*C*_i_) and transpiration rate (*T*_r_) (Fig. 1) for maintenance of photosynthetic capacity [60]. Conversely, the ALA-induced H_2_O_2_ accumulation in strawberry roots may be act as a necessary cell signal to regulate the related gene expressions, such as the upregulation of genes involved in Na^+^ absorption, transport, and distribution in response to salt stress [61]. It can be inferred that the H_2_O_2_ increase promoted by exogenous ALA may also regulate gene expression to withstand osmotic stress. Nevertheless, the underlying mechanism is still unclear up to now.

Here, the RNA-Seq data of strawberry leaves and roots under control, PEG, and PEG + ALA treatments can provide a transcriptome-level perspective to discover the clues at a high resolution. A total of 9315 DEGs were discovered among PEG and PEG + ALA treatments in leaves and roots at three different times (Fig. 3), for which the large-scale remarkable variations in gene expressions have also been reported in *P. wutunensis* after application of ALA under salt stress [17]. It is elucidated that ALA can exert an influence on a significant portion of gene expressions not only in the herbaceous strawberries but also in woody poplars for responding to environmental stresses. Subsequently, the further GO and KEGG enrichment of these ALA-induced DEGs preferred clustering in the terms associated with secondary metabolite synthesis, as well as plant hormone synthesis and signal transduction (Figs. 4 and 5), which were similarly detected in ALA treated poplars under salt damage [17] and tea trees under cold stress [62]. As widely reported so far, plants physiological and biochemical responses are through altering their secondary metabolites and phytohormones induced by abiotic stress, naturally including osmotic stress [20]. Therefore, the exogenous ALA can help the osmotic stressed plant cells quickly initiate acclimatization for osmotic equilibrium, desiccation tolerance, and antioxidant defense [63].

On the one hand, the secondary metabolites represent relatively lower concentration but extreme impact on plants suffering osmotic conditions [64–67], which were sustainably activated by ALA in strawberries, such as the DEGs relevant to biosynthesis of terpenes, phenolics, isoprenoids, alkaloids and phenylpropanoids (Figs. 4 and 5). It is worth noticing that the ALA activated the DEGs connected to terpene biosynthesis both appearing in leaves and roots at the early stage, in especial more DEGs of roots enriched in the KO of ‘Sesquiterpenoid and triterpenoid biosynthesis’ than those of leaves in this study (Table S5 and S6). The terpenes are the largest class among the important secondary metabolites defending ecological stresses. They play dominant roles in plant defense system [67], through serving as the membrane stabilizers to block proton leakage [68] or as the potent antioxidants to scavenge ROS [69, 70]. In this study, the roots of drought-sensitive strawberries are the first tissue to experience the osmotic imbalance of the matrix, and consequently, ALA may rapidly facilitate more accumulation of terpenoids in roots withstanding osmotic stress. For example, the GERDs (alias *VIT_19s0014g04930* in Table S6) were enriched in ‘Sesquiterpenoid and triterpenoid biosynthesis’, which were significantly up-regulated by exogenous ALA both in strawberry leaves and roots. A series of evidences have also indicated that transcript levels are positively parallelled with the accumulation of secondary metabolites in the ecological stressed plants, certainly including germacrene D, an essential oil exerting non-enzymatic antioxidative protection [71–73]. As the previously reported, the up-regulation of *GERS*/*GERD* genes and accumulation of sesquiterpenes was a osmotic stress dependent processing in basil (*Ocimum basilicum*) [71], as well as sesquiterpenes were proportionally enhanced in sweet wormwood (*Artemisia annua* L.) related to the drought severity [72]. It can be inferred that ALA is beneficial to germacrene D and other terpenes formation and accumulation to avoid oxidative damages in PEG-stressed strawberries. Furthermore, the ALA-induced upregulation of *PPOs* were enriched in the KOs of ‘Biosynthesis of secondary metabolites’ and ‘Isoquinoline alkaloid biosynthesis’, encoding polyphenol oxidase related to biosynthesis of aurone, nordihydroguaiaretic acid, and betalains [74]. For example, the alkaloid betalains protect plants from the adverse stresses as the nonenzymatic antioxidant substance for ROS-scavenging, and also as osmotic substances for osmotic regulation [75]. In *Disphyma australe*, salt stress elevated the betalain content in leaves contributing to amelioration of salinity tolerance [76]. These suggest that the induction of *PPOs* by ALA may be one of the modes to accelerate osmotic stress tolerance in strawberries. Besides, the up-regulation of *EBOS*, *TPS9* (encoding terpene synthase 9), *TKT2* (encoding 1-deoxy-D-xylulose-5-phosphate synthase, DXS, related to isoprenoid precursor biosynthesis [77]) further encourage that ALA universally stimulates terpenes and other secondary metabolic enrichment against osmotic stress.

On the other hand, ALA also promoted the expression levels of genes involved in phytohormones synthesis and signal transduction (Figs. 4 and 5), which contribute to a great deal of assistance in coping with various environmental injuries [78]. For example, *MYC2*, *GH3.5*, *AOS1*, *AOC*, *OPR3* and *JMT* were involved in the biosynthesis, catabolism and signaling of JAs, *NCED1* (9-cis-epoxycarotenoid dioxygenase), *SAPK2*/*SnRK2* and *PP2C*s in abscisic acid (ABA) biosynthesis and signaling, and *ACS1* (1-amino-cyclopropane-1-carboxylic acid synthase) and *ERF1B* (ethylene-responsive transcription factor) in ethylene signaling pathway (Table S5 and S6). The most impressive performance was detected among the ALA-induced DEGs related to lipid-derived JAs (free-acid JA, MeJA and JA-Ile) biosynthesis, catabolism and signaling, according to KEGG enrichment (Fig. 5) and temporal expression trends (Fig. 6), approximately half of these DEGs in the clusters positive sensitive to exogenous ALA in strawberry leaves and roots under osmotic stress. The JAs are important cellular mediators for ROS scavenging, root development, secondary metabolism and water balance in plant growth and development, as well as biotic and abiotic stress response [21]. In this study, the JA-related genes differentially expressed between PEG-treated and PEG + ALA co-treated strawberries, such as the early induction of JAs biosynthetic genes, *LOX2.1*, *AOS3*, *OPR3* and *JMT* (Fig. 7). It is agreed with that of ALA-treated *P. wutunensis* under salt stress, in which the gene expression of *LOX2S*, *AOC* and *OPR3* was also upregulated by exogenous ALA [17]. The expression levels of *AOC*, *AOS*, *JAZ*, and *OPR* genes were significantly increased in drought stressed *Pohlia nutans* (Antarctic moss), which was speculated that JA signaling was one of the important induced pathways to adapt to the harsh living conditions [79]. Under drought stress, the *MtLOX2*, *MtAOS* and *MtOPR* genes were more quickly and constantly induced in the drought-tolerant chickpea compared with those in the sensitive variety, giving rise to the synthesis and accumulation of JAs [80]. Therefore, the cultivated strawberry is a drought sensitive plant, whose slower and fainter induction of JAs biosynthetic genes can be enhanced by exogenous ALA. It seems that PEG did not affect JAs content in the strawberry, however, the content of JAs, such as JA, MeJA, and JA-Ile were rapidly increased in the leaves and roots of PEG-stressed strawberry after ALA treatment (Fig. 10). These suggest that ALA may quickly accelerate expression of these specific genes, contributing to JA and MeJA accumulation and exerting as a key mediation to alleviate the deleterious effects of ecological stresses [81]. Besides, the exogenous MeJA exhibited similar remission effect as ALA on strawberries under water deficit condition (Fig. 11), consisting with the previous reports that exogenous JAs reduced the harmful effects by enhancing the root growth and photosynthetic capability [82, 83]. In contrary, JA synthetic inhibitor DIECA further aggravated the osmotic stress injuries, while ALA ameliorated the adverse effect to a certain extent (Fig. 11). These results indicate MeJA is critical for the ALA-stimulated osmotic stress tolerance in strawberries, similar with the coactive relationship between ALA and JAs on salt tolerance of *P. wutunensis* [17] and low temperature resistance of tomato [16]. In addition, one of the mechanisms for JA-defending osmotic stress is that JAs promote water uptake from moisture deficit soils to regulate the hydraulic potential of root cells, potentially by increasing the expression of aquaporin genes and the phosphorylation state of aquaporins [84, 85]. Our findings further corroborated this perspective that the significant increasing of tissue RWC and aquaporin gene expressions of roots, observed in both exogenous MeJA and ALA treatments compared with the PEG treatment (Fig. 11). The similar yet less effectiveness of MeJA when compared to ALA in water transduction consequently reveals that MeJA potentially serves as a downstream and acts a dominate role in the ALA-mediated water absorption and transport pathway under osmotic stress. Moreover, the ALA motivated JA and MeJA production might further result in activation of JA signaling pathway, reflecting by the significant up-regulation of *JARs*, *MYC2s*, *MED25*, *GL3s* and *ICE* in PEG-stressed strawberries, but relatively smaller increasing amplitude in *JAZ* transcripts (Fig. 7). Similarly, the ALA treatment also upregulated the *JAR1*, *MYC2* and *JAZ* gene expression in salt-stressed *P. wutunensis* under salt stress, might be the feedback of ALA-stimulated JAs accumulation to activate the JAs signaling channel [17]. As widely known, the JAR catalyzes JA into JA-Ile, a higher bioactive substance, which perceives and promotes the complex of JA receptor F-box protein SCF^COI1^ and JAZ repressor protein, and then leads to JAZ degradation dependent on SCF^COI1^ and 26 S proteasome [22]. Therefore, exogenous ALA may consequently relieve the JAZ inhibition on the transcription of JAs responsive genes for signaling cascade, such as the release of *ICE*s for freezing tolerance [23] and *GL3s* withstanding insect and herbivores in *Arabidopsis* [25]. Notably, MYC2, the central coregulator in JAs signaling, can also be released from JAZ repression and subsequently activate downstream genes involved in abiotic stress tolerance by forming a transcriptional activation complex with the Mediator transcriptional coactivator MED25 subunit [24, 86]. Overexpression of *Camellia sinensis CsMYC2* in *Arabidopsis* enhanced the osmotic stress caused by mannitol, along with the JA content improvement, while suppression expression of *CsMYC2* in tea plant led to the opposite results [87]. A feedback loop of CsMYC2-*CsLOX7*/*CsAOS2* was proposed through CsMYC2 positively binding with the promoters of *CsLOX7* and *CsAOS2* for JA accumulation to resist to osmotic stress in tea plant [87]. Therefore, all of these suggest that ALA may enhance the JA-JAZ-MYC2 signaling switch to initiate the signal transduction cascades in response to osmotic stress, in which the activated MYC2 might act as the hub role in JA pathway.

In plants, crosstalk of JAs with other important hormones and secondary metabolites has been revealed at the hormone response and biosynthesis level, which plays a key role in various responses to environmental stimuli [18–20]. In our study, a comprehensive insight into ALA shaping the JA-related cross-regulatory relationships in osmotic stressed strawberries at the transcriptional level, that is, the co-expression networks exhibited by the hub genes from darkmagenta and mediumpurple3 modules (Fig. 9). For example, the *SAPK2*/*SnRK2* whose protein kinase SnRK2 can active ABA signaling transduction after separating from phosphatase PP2C [88], was classified in darkmagenta module and co-expressed with *GH3.5*. However, the *SAPK2*/*SnRK2* (augustus_masked-Fvb1-4-processed-gene-113.7) was indistinctive up-regulated or down-regulated by ALA in leaves and roots, which might block the ABA signaling response under osmotic stress, such as regulating stomatal closure. As we previously reported that ABA-stimulated stomatal closure can be inhibited by exogenous ALA through ALA-induced PP2A activity, subsequently lead to SnRK2.6 dephosphorylation and lower kinase activity in apple leaves under normal condition [60]. Our present results that the PEG stress caused decreasing stomatal conductance, while ALA relieved the effect further support the inference at physiological level (Fig. 1). Additionally, the ALA-promoted crosstalk signaling pathway between JA and secondary metabolites is potentially dependent on the co-expression of *LOX2.1* with *GERD* and *EBOS* in mediumpurple3 module (Fig. 9). Notably, in the aspect of *GERD* gene (also named as *FaTPS1*), its expression level was also up-regulated by exogenous MeJA and its overexpression enhanced the emission of germacrene D as well as other terpenes, which contributed to resistance improvement against *Botrytis cinerea* infection in strawberry fruits [89]. Furthermore, the more direct evidence of JA and terpenes interaction was the FaMYC2, as the important JA signaling hub, bound with the promoter of the *GERD* gene to facilitate terpenoids synthesis and downstream cascade [89]. Combined with our above-mentioned of terpenes in abiotic stress, all of these indicate that JAs may be the intermediary roles for ALA signaling pathway in terpene accumulation for osmotic resistance.

**Fig. 12:**
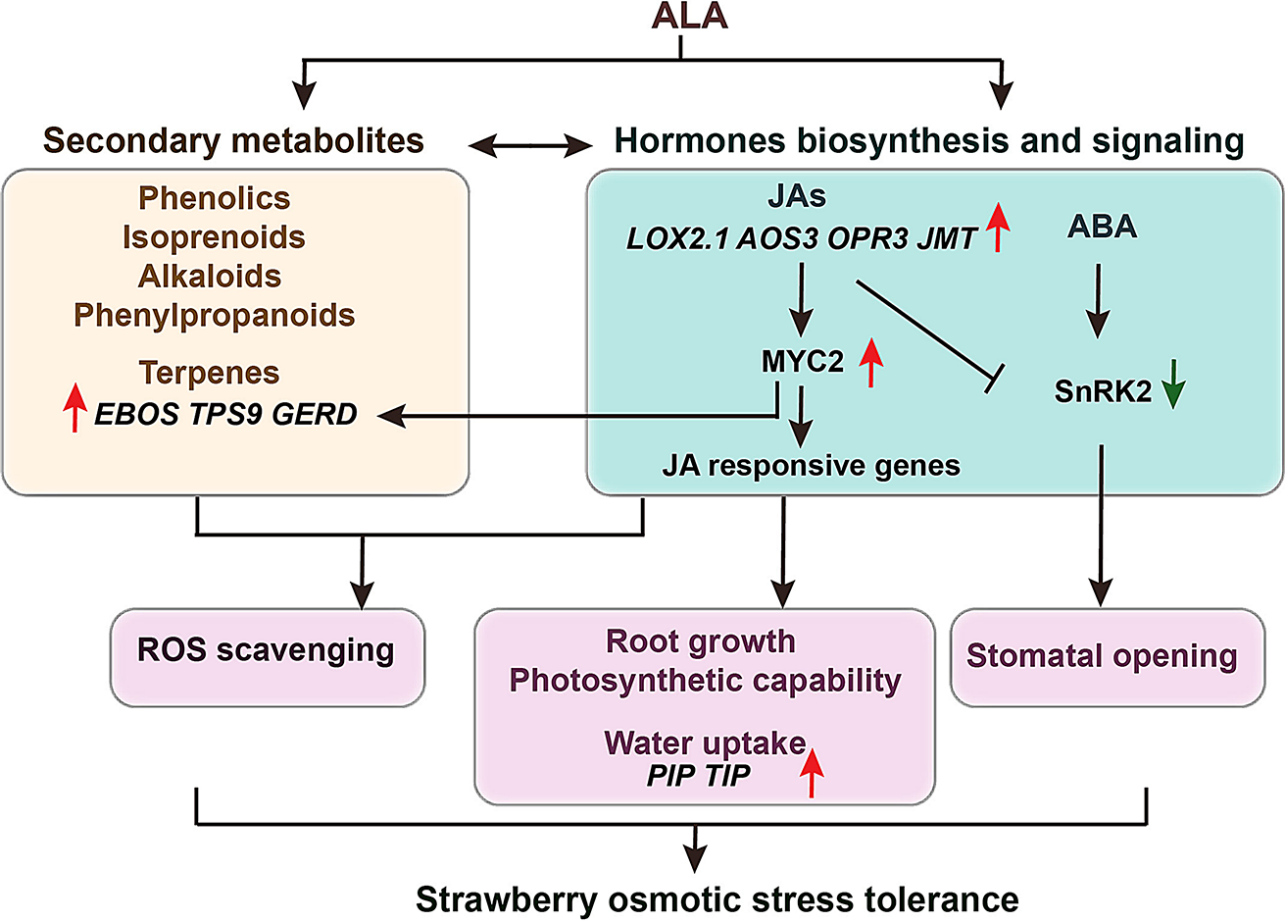
Putative schematic model of ALA improves osmotic stress tolerance in strawberries. The red arrow means up-regulation, and the green arrow means down-regulation

In conclusion, we proposed a potential model of exogenous ALA induced osmotic stress tolerance in strawberries (Fig. 12). This model provides two main approaches of secondary metabolites accumulation and hormones biosynthesis and signaling activation by regulating the transcriptional levels of key genes to counter drought injuries, when strawberry plants were treated with ALA. Notably, in the aspect of hormones, the ALA-stimulated JAs synthesis and signaling not only regulate JA responsive genes but also carry out the crosstalk with terpenes or ABA transduction. The elevated ALA-JA pathway is beneficial to oxidative defense enhancement by scavenging ROS, the aqueous homeostasis maintenance by root water uptake, root growth as well as the root growth and leaf photosynthetic capability improvement, thereby improving the osmotic tolerance in strawberries. Therefore, it is proposed that JAs play important roles in mediating ALA-advanced plant drought tolerance.

## Conclusions

In this study, exogenous ALA enhanced the RWC, root development, gas exchange parameters and antioxidant enzyme activities, and decreasing the leaf H_2_O_2_ and MDA content of cultivated strawberries under osmotic stress, suggesting that ALA can improve the osmotic tolerance of strawberries. The transcriptome analysis showed that the DEGs sensitive to exogenous ALA were involved in the secondary biosynthesis and hormones signaling pathways, in which they were especially related to JAs biosynthesis and signaling. ALA promoted JA-Ile accumulation in the strawberry leaves and roots under osmotic stress. In addition, there was similar mitigative effect between exogenous MeJA and ALA on strawberries exposed to osmotic stress, but the negative effect caused by exogenous DIECA can be relieved by ALA. In general, our findings suggest that JAs might play important roles in ALA-induced osmotic tolerance, which can also provide a novel sight to uncover the mechanism of ALA improving osmotic tolerance of plants.

## Electronic supplementary material

Below is the link to the electronic supplementary material.

Supplementary Material 1: Table S1. The primers for qRT-PCR analysis.

Supplementary Material 2 :Table S2. Statistics of RNA-seq reads for the 54 samples.

Supplementary Material 3: Table S3.The mapping statistics for the 54 samples.

Supplementary Material 4: Table S4.Pearsons correlation between RNA-seq and qRT-PCR results for ten DEGs.

Supplementary Material 5: Table S5. Top 15 GO classes of the enriched GO analysis in different treatments.

Supplementary Material 6: Table S6. Top 15 KO pathways of the enriched KEGG analysis in different treatments.

Supplementary Material 7: Table S7. The FPKM values of JA-related genes in strawberries under different treatments.

Supplementary Material 8: Table S8. The expression clusters of JA-related genes in strawberry leaves and roots.

Supplementary Material 9: Table S9. Top 15 GO enrichment in 17 modules.

Supplementary Material 10: Table S10. Top 15 KEGG enrichment in 17 modules.

Supplementary Material 11: Table S11. The correlation of JA-related gene expressions and modules.

## Data Availability

The raw datasets of transcriptome sequencing are available in the NCBI repository, PRJNA1144869.

## Abbreviations

ALA: 5-Aminolevulinic acid
WGCNA: Weighted Gene Correlation Network Analysis
PEG: Polyethylene Glycol 6000
RWC: Relative Water Content
MDA: Malondialdehyde
DEG: Differentially Expressed Gene
JA: Jasmonate
MeJA: Methyl Jasmonate
JA-Ile: (+)-7-Iso-jasmonoyl-L- isoleucine
PSII: Photosystem II
SCF^COI1^: Skp/Cullin/F-box CORONATINE INSENSITIVE1
TF: Transcription Factor
DIECA: Diethyldithiocarbamate
RNA-seq: RNA-sequencing
FW: Fresh Weight
DW: Dry Weight
SW: Saturated Weight
SOD: Superoxide Dismutase
POD: Peroxidase
CAT: Catalase
FPKM: Fragment per kilobase of transcript per million reads
GO: Gene Ontology
KEGG: Kyoto Encyclopedia of Genes and Genomes
